# Conversion of Transplanted Mature Hepatocytes into *Afp* ^+^ Reprogrammed Cells for Liver Regeneration After Injury

**DOI:** 10.1002/advs.202517126

**Published:** 2026-01-29

**Authors:** Ting Fang, Chao Yang, Hua Qiu, Yuan Du, Xicheng Wang, Yuting Li, Mingyang Xu, Changcheng Liu, Xiuhua Li, Na Guo, Jun Shi, Wencheng Zhang, Zhiying He

**Affiliations:** ^1^ Institute for Regenerative Medicine Medical Innovation Center and State Key Laboratory of Cardiovascular Diseases School of Life Sciences and Technology Shanghai East Hospital Tongji University Shanghai P. R. China; ^2^ Shanghai Engineering Research Center of Stem Cells Translational Medicine Shanghai P. R. China; ^3^ Shanghai Institute of Stem Cell Research and Clinical Translation Shanghai P. R. China; ^4^ The First Affiliated Hospital of Nanchang University Nanchang Jiangxi P. R. China; ^5^ Hubei Provincial Clinical Research Center for Umbilical Cord Blood Hematopoietic Stem Cells Hubei Key Laboratory of Embryonic Stem Cell Research Taihe Hospital Hubei University of Medicine Shiyan Hubei P. R. China

**Keywords:** alpha‐fetoprotein‐positive reprogrammed hepatocytes (*Afp* ^+^ rHeps), hepatocyte transplantation, liver regeneration, PPARγ pathway, TNF‐α/AP‐1 signaling

## Abstract

Hepatocyte transplantation effectively treats liver failure, yet the regenerative mechanisms driven by engrafted mature hepatocytes remain elusive. Through integrated serial transplantation, lineage tracing, single‐cell RNA sequencing (scRNA‐seq), and single‐cell transposase‐accessible chromatin sequencing (scATAC‐seq), we show that donor hepatocytes convert into transitional, alpha‐fetoprotein‐positive reprogrammed hepatocytes (*Afp^+^* rHeps). These cells exhibit controlled proliferation while maintaining unipotent hepatic differentiation potential, enabling fully functional maturation after rapid expansion. Such plasticity is dynamically regulated by AFP expression level‐dependent metabolic remodeling through the peroxisome proliferator‐activated receptor γ (PPARγ) pathway, which coordinates two functionally distinct subpopulations: *Afp* ^low^ cells sustain proliferation by activating energy metabolism pathways, whereas *Afp* ^high^ cells adapt to stress by switching to β‐oxidation. Additionally, the proliferation of *Afp^+^* rHeps is driven and sustained by tumor necrosis factor‐alpha (TNF‐α)/activator protein‐1 (AP‐1) signaling derived from host liver neutrophils. Spatiotemporal analysis further shows that transforming growth factor‐beta (TGF‐β)‐mediated migration precedes PPAR‐driven metabolic zonation, ensuring ordered niche adaptation. Together, these findings delineate the molecular basis of liver regeneration mediated by transplanted mature hepatocytes and pinpoint the PPARγ/AFP metabolic axis and TNF‐α/AP‐1 mitogenic signaling as actionable levers to optimize regenerative therapies based on terminally differentiated hepatocytes.

## Graphical abstract

Donor‐derived tdTomato^+^ mature hepatocytes were FACS‐isolated and transplanted into *Fah* ^−/−^ host mice. During regeneration, these cells convert into proliferative, unipotent *Afp^+^* rHeps. Their plasticity is governed by a PPARγ/AFP‐dependent metabolic switch, segregating into pro‐proliferative *Afp* ^low^ and pro‐survival *Afp* ^high^ subpopulations. Neutrophil‐derived TNF‐α/AP‐1 signaling drives and sustains *Afp^+^* rHep proliferation. Over the course of repopulation, progressive reconstruction of liver zonation is achieved.

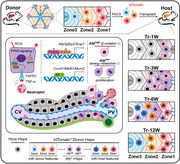

**Abbreviations**
AFP: alpha fetoprotein
*Afp* ^+^ rHep: alpha‐fetoprotein‐positive reprogrammed hepatocyte
ALF: acute liver failure
ALT: alanine aminotransferase
AP: average precision
AP‐1: activator protein‐1
APAP: acetaminophen
ARS: *Afp* ^+^ rHep‐related signature
AST: aspartate aminotransferase
AUC: area under curve
BEC: biliary epithelial cell
CCl_4_
: carbon tetrachloride
cDC: conventional dendritic cell
Co‐IP‐MS: co‐immunoprecipitation coupled with mass spectrometry
CV: central vein
DAP: differential accessible peak
DC: diffusion component
DDC: 3,5‐diethoxycarbonyl‐1,4‐dihydrocollidin
DDR: DNA damage response
DEG: differentially expressed gene
DTW: dynamic time warping
ECM: extracellular matrix
EMP: epithelial‐mesenchymal plasticity
EMT: epithelial‐mesenchymal transition
ETC: electron transport chain
FA: fatty acid
FACS: fluorescence‐activated cell sorting
FC: fold change
FDR: false discovery rate
GO: Gene Ontology
GRN: gene regulatory network
GSEA: gene set enrichment analysis
GSVA: gene set variation enrichment analysis
H&E: hematoxylin‐eosin
HCC: hepatocellular carcinoma
HPCs: hepatic progenitor cells
HSC: hepatic stellate cell
HSR: heat shock response
HySR: hypoxia stress response
IF: immunofluorescence
IHC: immunohistochemistry
IL‐6: interleukin‐6
IVC: inferior vena cava
KC: kupffer cell
KEGG: Kyoto Encyclopedia of Genes and Genomes
kME: module eigengene‐based connectivity
LPLC: liver progenitor‐like cell
LSEC: liver sinusoidal endothelial cell
MFI: mean fluorescence intensity
MoMF: mononuclear‐derived macrophage
mtDNA: mitochondrial DNA
nDNA: nuclear DNA
NES: normalized enrichment score
NMF: non‐negative matrix factorization
NPC: non‐parenchymal cell
NTBC: 2‐(2‐nitro‐4‐trifluoro‐methylbenzyol)‐1,3‐cyclohexanedione
OSM: oncostatin‐m
OSR: oxidative stress response
OXPHOS: oxidative phosphorylation
PC_LVEC: pericentral liver vascular endothelial cell
PCA: principal component analysis
pDC: plasmacytoid dendritic cell
PHC: primary hepatocyte
PHx: partial hepatectomy
PP_LVEC: periportal liver vascular endothelial cell
PPAR: peroxisome proliferator‐activated receptor
PPI: protein‐protein interaction
PPP: pentose phosphate pathway
PPRE: peroxisome proliferator response element
PR: precision‐recall
PV: portal vein
qPCR: quantitative polymerase chain reaction
rAFP: recombined AFP
ROC: receiver operating characteristic
ROS: reactive oxygen species
RRG: reprogramming‐related gene
RRP: regeneration response program
RSS: relative specificity score
scATAC‐seq: single‐cell transposase‐accessible chromatin sequencing
scRNA‐seq: single‐cell RNA sequencing
TBG: thyroxine binding globulin
Tbil: total bilirubin
TCA: tricarboxylic acid
TF: transcription factor
TFBS: transcription factor binding site
TG: triacylglycerol
TGF‐β: transforming growth factor‐beta
TNFR: TNF‐α receptor
TNF‐α: tumor necrosis factor‐alpha
TOM: topological overlap matrix
TSS: transcription start site
UMAP: uniform manifold approximation and projection
UMI: unique molecular identifier
UPR: unfolded protein response
WB: western blot
WT: wild‐type

## 1 Introduction

Various injurious stimuli (e.g., viral infections, alcohol abuse, toxins) induce hepatocyte death (apoptosis or necroptosis), driving progression of liver fibrosis, cirrhosis, and cancer. Tissue repair after injury primarily relies on compensatory proliferation of residual quiescent hepatocytes, which are reactivated by inflammatory cytokines such as interleukin‐6 (IL‐6) and tumor necrosis factor‐alpha (TNF‐α) to re‐enter the cell cycle [1, 2, 3, 4, 5, 6, 7, 8]. However, the molecular mechanisms governing hepatocyte reactivation from quiescence remain incompletely understood.

Hepatocyte proliferation depends on intrinsic plasticity. Mature hepatocytes retain accessible chromatin regions of hepatic progenitor genes, endowing them with reprogramming potential to rapidly respond to injury [9]. In 3,5‐diethoxycarbonyl‐1,4‐dihydrocollidin (DDC)‐induced periportal injury, IL‐6/STAT3 signaling drives hepatocyte conversion into liver progenitor‐like cells (LPLCs) by reactivating *Sox9*, *Spp1*, and *Sox4* [10]. In acetaminophen (APAP)‐induced perivenular injury, hepatocytes at the interface between damaged and non‐damaged tissue upregulate fetal‐specific genes (e.g., *Afp*, *Cdh17*) [11]. Partial hepatectomy (PHx) studies confirm hepatocytes undergo “postnatal‐like reprogramming”, transiently reactivating an early‐postnatal gene program to enable proliferation [12]. Notably, transplantation experiments demonstrate donor hepatocytes similarly expand by reprogramming into hepatic progenitor cells (HPCs), supporting a conserved mechanism [13]. This developmental analogy leads to a model wherein liver regeneration involves mature hepatocytes reacquiring a transient, proliferative yet functional state, similar to that of immature postnatal hepatocytes, which possess both basic function and active proliferative capacity before full maturation [14, 15, 16, 17].

Our previous study demonstrated transplanted mature hepatocytes exhibit remarkable proliferative potential, sustaining regenerative capacity through 12 rounds of serial transplantation in fumarylacetoacetate hydrolase deficiency (*Fah* ^−/−^) mice (69 cell divisions) [18]. Building on this, we developed in vitro systems to expand hepatocytes via reprogramming or transdifferentiation, addressing donor cell shortages for transplantation [19, 20, 21, 22, 23]. These findings set the stage to investigate how donor hepatocytes reprogram and proliferate within the injured host microenvironments. Hepatocyte transplantation holds great promise for metabolic and end‐stage liver diseases, but clinical translation is hampered by short‐lived therapeutic effects, low engraftment efficiency, unclear donor cell fate regulation, and immune rejection. Understanding donor hepatocyte proliferation biology is key to overcoming these hurdles, with models like *Fah* ^−/−^ and urokinase‐type plasminogen activator (uPA) transgenic mice enabling robust expansion of transplanted cells for mechanistic dissection [24]. Notably, effective liver repair requires hepatocytes to balance proliferation with specialized functions [9, 10, 25, 26, 27, 28]. In injury models wherein hepatocyte loss is the dominant feature (e.g., PHx or *Fah* ^−/−^ mice), this balance is achieved through partial regression to a precursor‐like state, rather than full dedifferentiation.

Three critical gaps remain unresolved. First, the precise intermediate cellular states of transplanted hepatocytes during repopulation are uncharacterized, despite evidence of reprogramming in *Fah* ^−/−^ mice [13]. Second, it remains unclear how transplanted hepatocytes balance proliferation with metabolic function, a prerequisite for effective repair [29, 30]. Third, the host‐derived signals in the injured microenvironment that spatiotemporally regulate donor cell fate (beyond general pro‐proliferative cytokines like IL‐6/TNF‐α [7, 31]) are undefined. By framing these interconnected gaps, we provide a compelling rationale for our study aims: to identify and characterize the *Afp* ^+^ rHep state, dissect its dual metabolic and proliferative modules, and decode the host‐derived signaling axis regulating it.

While proliferating hepatocytes are barely accessible in PHx models, those in *Fah* ^−/−^ mice can be efficiently isolated. Here, using cell tracing, single‐cell RNA sequencing (scRNA‐seq), and single‐cell transposase‐accessible chromatin sequencing (scATAC‐seq), we dissected the cellular state changes, regional properties, fate transitions, and regulatory mechanisms of transplanted mature hepatocytes during liver regeneration. Specifically, we demonstrated that host liver injury activates regeneration, prompting transplanted hepatocytes to reprogram into alpha‐fetoprotein (AFP)‐positive reprogrammed hepatocytes (*Afp* ^+^ rHep), which promote repair via proliferation and re‐maturation.

## 2 Results

### 2.1 Transplanted Mature Hepatocytes Acquire Proliferative Capacity through Reprogramming

To investigate how transplanted mature hepatocytes regenerate injured livers, we prepared tdTomato^+^ donor hepatocytes by intravenously delivered AAV8‐TBG‐Cre to Rosa26‐LSL‐tdTomato mice for hepatocyte‐specific tdTomato labeling, followed by fluorescence‐activated cell sorting (FACS) (Figure S1A–D). The purified mature hepatocytes were then transplanted into *Fah* ^−/−^ mice via intrasplenic injection, followed by withdrawing daily 2‐(2‐nitro‐4‐trifluoro‐methylbenzyol)‐1,3‐cyclohexanedione (NTBC) administration of hosts. Without NTBC, *Fah* ^−/−^ mice develop irreversible hepatocyte damage due to tyrosine catabolism defects. Transplanted mature hepatocytes engrafted and proliferated rapidly in injured livers, effectively repopulating the liver and restoring its architecture and function (Figure S1E–I).

**FIGURE 1:**
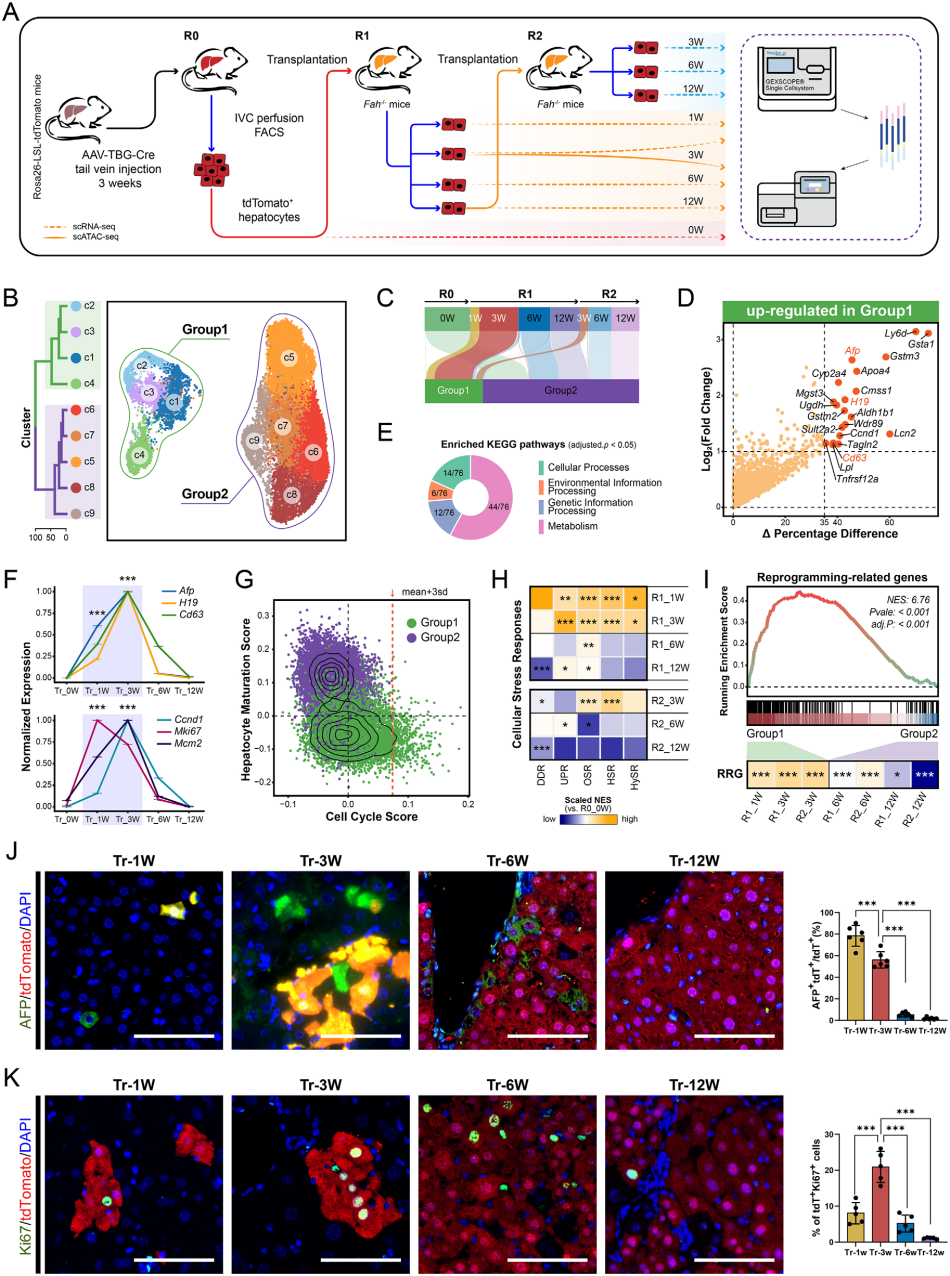
Transplanted mature hepatocytes are reprogrammed at the early stage of liver regeneration. Additional details are provided in Table S1. (A) Schematic overview of the experimental strategy: serial transplantation of tdTomato^+^ hepatocytes, followed by single‐cell sequencing of tdTomato^+^ hepatocytes isolated from host livers at different timepoints post‐transplantation. (B) Uniform manifold approximation and projection (UMAP) of all 29,130 tdTomato^+^ hepatocytes from eight samples, colored by cell clusters. Green and purple circles outline Group1 and Group2, respectively, as defined by hierarchical clustering (**left**). (C) Stream plot depicting the dynamic proportion distribution of Group1 and Group2 cells across eight samples. The width of each stream corresponds to the proportion of each group at different timepoints. (D) Volcano plot based on the percentage difference (x‐axis) and the log_2_(fold change) (log_2_FC, y‐axis) shows the upregulated genes in Group1 relative to Group2 cells. Genes highlighted in orange are important for identifying cellular states. (E) Donut chart showing the proportion of the four major categories of the Kyoto Encyclopedia of Genes and Genomes (KEGG) pathways enriched by the upregulated genes in Group1 relative to Group2 cells (adjusted *p* < 0.05). (F) Line charts display the average expression levels of three hepatic progenitor genes (**top**) and three cell cycle genes (**bottom**) over eight samples. Sample points represent the mean expression levels of all cells in a sample, error bars show the mean ± SEM, and segment bandwidths depict the confidence intervals. Asterisks mark significantly upregulated genes in each comparison (Tr_1 W vs. Tr_0 W and Tr_3 W vs. Tr_0 W; two‐sided Wilcoxon rank sum test, ^***^adjusted *p* < 0.001). (G) Scatter plot of the "Cell Cycle Score" (x‐axis) against the "Hepatocyte Maturation Score" [17] (y‐axis). Green and purple dots correspond to Group1 and Group2 cells, respectively. The orange dotted line denotes the threshold set at the mean Cell Cycle Score plus three standard deviations, and cells above this threshold are classified as cycling cells. (H) Heatmap showing the average normalized enrichment score (NES) of pathways related to cellular stress response [95] for each transplant timepoint relative to R0_0 W. Asterisks denote pathways that are significantly different from R0_0 W (^*^adjusted *p* < 0.05, ^**^adjusted *p* < 0.01, ^***^adjusted *p* < 0.001). (I) Gene set enrichment analysis (GSEA) of reprogramming‐related genes (RRG) [10] in Group1 vs. Group2 cells (**top**). Average NES of RRG for each transplant timepoint relative to R0_0 W (**bottom**). Asterisks mark a significant difference from R0_0 W (^*^adjusted *p* < 0.05, ^***^adjusted *p* < 0.001). (J,K) Immunofluorescence (IF) staining of tdTomato and AFP (J) or Ki67 (K), respectively, in host livers at four timepoints post‐transplantation (1 W, 3 W, 6, and 12 W) (**left**). The ratios of tdTomato^+^ AFP^+^ (J) or tdTomato^+^ Ki67^+^ (K) hepatocytes were quantified, respectively (shown as the bar chart to the **right** of each set of images). Scale bars: 200 µm. ^***^ *p* < 0.001.

We implemented a two‐round serial transplantation strategy: donor‐derived hepatocytes were FACS‐sorted from primary recipient livers and re‐transplanted into new *Fah* ^−/−^ recipients using identical protocols to assess their repopulation capacity (Figure 1A). Hepatocytes were isolated at five chronological phases from: (1) First‐round transplantation: tdTomato^+^ primary hepatocytes (R0_0 W) transplanted into *Fah* ^−/−^ mice, with sampling at 1/3/6/12 weeks post‐transplantation (R1_1 W to R1_12 W); (2) Second‐round transplantation: Fully repopulated hepatocytes (R1_12 W, Figure S1E) were retransplanted, with sampling at 3/6/12 weeks (R2_3 W to R2_12 W). All samples underwent scRNA‐seq.

Following standard quality control (Figure S2A), we focused on tdTomato^+^ cells to track donor hepatocytes and their progeny. Data from matched time points across the two transplantation rounds showed strong concordance (Figure S2B,C), indicating conserved regenerative patterns during serial transplantation. Clustering analysis (Figure S2B) revealed that: early‐phase cells (1‐3 weeks (W) post‐transplantation) exhibited significant transcriptomic divergence from primary hepatocytes (0 W); mid‐late phase cells (6‐12 W), particularly those at 12 weeks, progressively regained primary hepatocyte characteristics.

After stringent quality control and standardized processing, 29,130 high‐quality cells were classified into 9 transcriptionally distinct clusters (c1‐c9), which coalesced into two well‐segregated populations (provisionally termed Group1 and Group2; Figure 1B). Cellular composition analysis revealed striking temporal partitioning: Group1 predominantly contained early‐repopulation cells (92.68% from 1 to 3 W post‐transplantation), whereas Group2 comprised mostly later‐phase cells (98.35% from 6 to 12 W) (Figure 1C; Figure S2D). The minimal stress index score [32] in R0_0 W controls (Figure S2E) excluded tissue dissociation‐induced hepatocyte damage. These data demonstrate that transplanted mature hepatocytes undergo extensive transcriptional remodeling during early engraftment, generating a transient subpopulation (Group1) with stage‐specific molecular signatures.

Transcriptomic analysis revealed transitional hepatocytes (Group1) highly expressed hepatic progenitor markers (*Afp*, *H19*, *Cd63*) [33, 34] (Figure 1D) compared to mature hepatocytes (Group2), with upregulated genes enriched in Cell cycle and metabolic reprogramming (Figure 1E; Figure S2F). These signature genes emerged by 1 week post‐transplantation (Tr_1 W; Figure 1F). Consistently, tdTomato^+^AFP^+^ cells peaked at Tr_1 W (∼90%), declining progressively to < 5% by Tr_12 W (Figure 1J; Figure S3A). Functionally, transitional cells exhibited elevated proliferative activity (Figure 1G) and accounted for the majority of cycling cells during early repopulation, with sustained expression of cell cycle genes (*Ccnd1*, *Mki67*, *Mcm2*; Figure 1F; Figure S2G). Notably, tdTomato^+^Ki67^+^ cell frequency peaked at Tr_3 W (Figure 1K; Figure S3B), suggesting a phased transition from fate commitment (Tr_1 W) to proliferative expansion (Tr_3 W).

In the injured liver microenvironment of *Fah* ^−/−^ mice, transitional cells (Group1) exhibited pronounced stress adaptation. Compared to mature hepatocytes (R0_0 W), they specifically upregulated antioxidant genes (*Gsta1*, *Gstm3*, *Mgst3*; Figure 1D) and exhibited extensive stress responses, with oxidative stress response (OSR) activation being the most prominent (Figure 1H). This pattern mirrored hepatocyte responses in DDC‐induced injury [27], suggesting conserved adaptation mechanisms may across injury models. Notably, reprogramming‐related genes (RRGs) [10] were significantly enriched in transitional cells compared to mature populations (Tr_6‐12 W; Figure 1I). Mechanistically, oxidative stress may drive transitional features through specific gene reprogramming, enhancing microenvironmental adaptability while acquiring proliferative capacity. This reprogramming was reflected in a systematic shift in gene module activity over time, where maturation signatures increased as stress/proliferation modules declined (Figure S2H), outlining a unified path to maturity.

In conclusion, upon the engraftment, reprogramming happened to the transplanted mature hepatocytes. And these reprogrammed hepatocytes were then further driven by the injured liver microenvironment to start repair and repopulation of the host liver via transitional features including metabolic remodeling and enhanced proliferation.

### 2.2 Transitional *Afp* ^+^ Reprogrammed Hepatocytes (*Afp* ^+^ rHeps) Exhibit are Partially Transcriptional Resemblance to Immature Hepatocytes and Are Functionally Distinct from Injury‐Induced LPLCs

**FIGURE 2:**
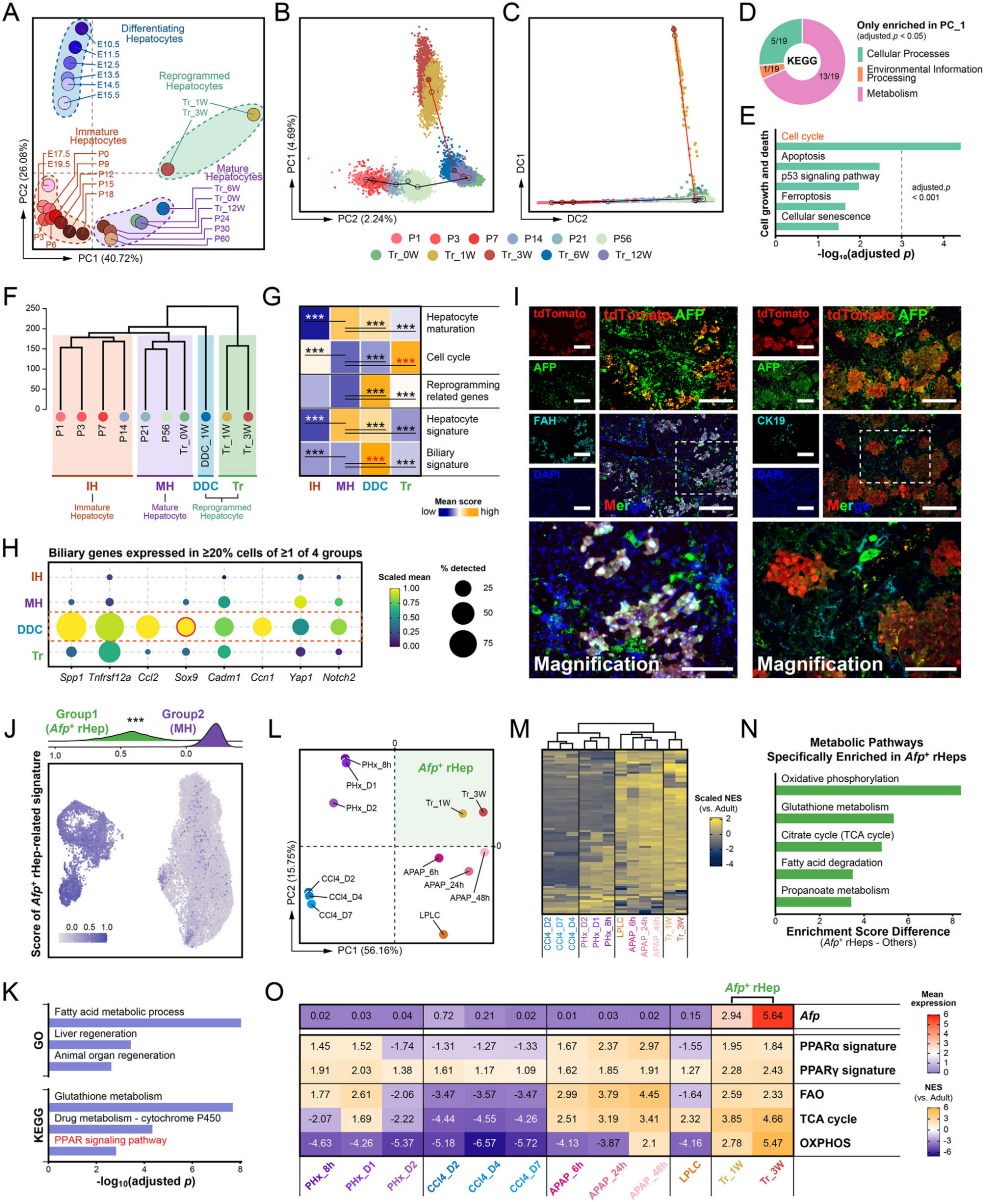
Metabolic remodeling and proliferation activation occur during the reprogramming of donor hepatocyte. Additional details are provided in Table S2. (A) Principal component analysis (PCA) plot based on maturation‐related genes [17] encompassing 23 discrete timepoints across liver development and regeneration. The dataset includes fetal (E10.5‐E19.5) [14, 17], postnatal (P0‐P60) [17], and transplanted (Adult/Tr_0W‐Tr_12 W) hepatocytes. Points are colored by their respective timepoint. Dotted lines in different colors outline the four major cell groups. (B‐C) PCA plot (B) and Diffusion map (C) each visualize the developmental progression of hepatocytes across 11 timepoints, including postnatal (P1‐P56) [12, 35] and transplanted (Adult/Tr_0W‐Tr_12 W) stages. In both panels, cells are colored by timepoint. Inferred trajectories for maturation (black) and transplantation (red) are overlaid. (D) Donut chart showing the proportion of KEGG pathways enriched exclusively by the PC1‐related genes identified in Figure 2B (adjusted *p* < 0.05). (E) Bar plot of KEGG pathways within the "Cell growth and death" category in Figure 2D. The x‐axis represents the ‐log_10_(adjusted *p*) from the enrichment analysis. (F) Hierarchical clustering of hepatocytes across 12 timepoints segregated them into four groups based on global gene expression profiles. The 12 timepoints include the DDC_1 W sample [10], which was added as a distinct timepoint for comparison with the analyses in Figure 2B,C. (G) Average module scores for gene sets characterizing hepatocyte states, shown for each group defined in Figure 2F. Asterisks denote gene sets that are significantly different from the MH group (two‐sided Wilcoxon rank sum test, ^***^adjusted *p* < 0.001). (H) Dot plot showing the scaled expression (color intensity) and cellular percentage (dot size) of biliary genes [96] that are expressed in ≥20% of cells in any group across the clusters defined in Figure 2F. (I) Multiplex IF staining for tdTomato, AFP, and FAH (**left**) or CK19 (**right**) in host livers at 3 weeks post‐transplantation. Scale bars:100 µm. (J) UMAP plot (**bottom**) shows the module scores of the *Afp* ^+^ rHep‐related signature (ARS) for each cell. The ridge plot (**top**) shows the distribution of these scores for both cell groups. (K) Bar plot of the selected Gene Ontology (GO) and KEGG terms enriched for the ARS (adjusted *p* < 0.01). The x‐axis represents the ‐log_10_(adjusted *p*) of the enriched terms. (L) PCA plot of metabolic pathway enrichment scores in hepatocytes during early regeneration. Timepoints (n = 12) were selected based on clustering patterns in Figure S4H, including PHx [97], CCl_4_ [61], and APAP [98] models (3 timepoints each), transplanted hepatocytes (2 timepoints), and LPLCs [27]. Samples are colored by timepoint, revealing the exclusive localization of transplanted *Afp* ^+^ rHeps (Tr_1 W, Tr_3 W) in Quadrant I, indicative of their unique metabolic state. (M) Heatmap of NES for 82 KEGG metabolic pathways across the 12 regeneration samples. Color intensity indicates NES, revealing pathway‐level details underlying the PCA patterns in Figure 2L. (N) Top 5 metabolic pathways with the highest specificity in *Afp* ^+^ rHeps. Bar plot shows the difference in mean enrichment scores between transplanted *Afp* ^+^ rHeps (Tr_1 W, Tr_3 W) and other regeneration hepatocytes (PHx, CCl_4_, APAP, LPLC) for pathways identified in Quadrant I of the pathway‐level PCA (Figure S4L). Positive values indicate *Afp* ^+^ rHeps‐specific enrichment. (O) Coordinated activation of energy metabolism in *Afp* ^+^ rHeps. Heatmap of *Afp* gene expression (mean) and NES for six core pathways shows specific upregulation of PPAR signaling (α/γ) [44], fatty acid oxidation (FAO) [99], TCA cycle, and oxidative phosphorylation (OXPHOS) in transplanted *Afp* ^+^ rHeps (Tr_1 W, Tr_3 W), indicating enhanced mitochondrial energy generation concurrent with high *Afp* expression.

To characterize transitional cells (Group1), we integrated transplanted hepatocyte data with murine liver developmental atlases (GSE90047 [14], GSE209749 [17]). Subsequent principal component analysis (PCA) was performed using a published hepatocyte maturation gene set (Clusters A‐D) [17]. The analysis revealed that transitional cells (Tr_1W‐3 W) formed a distinct cluster outside the continuum from E10.5 to adulthood (R0_0 W) (Figure 2A). Notably, these cells retained metabolism‐related gene expression (Clusters B‐D) while reactivating biosynthetic genes (Cluster A; including *Afp* and *H19*), selectively mimicking postnatal hepatocyte (P3‐P12) profiles (Figure S4A,B).

To rule out technical bias, we validated our findings using independent single‐cell datasets of postnatal hepatocytes (GSE151309 [12], GSE171993 [35]). PCA confirmed that transitional cells diverged from the PC2‐represented normal developmental trajectory, showing specific distribution along PC1 axis (Figure 2B). Notably, beyond metabolic regulation, PC1‐associated genes were significantly enriched in pathways related to Cell cycle, p53 signaling pathway, and Ferroptosis (Figure 2D,E), collectively indicating coordinated metabolism‐proliferation regulation. Diffusion map analysis (cell cycle effects excluded) similarly revealed transitional cells distributed along the diffusion component (DC) 1 axis, clearly segregated from the DC2‐based maturation path (Figure 2C).

Given the significant enrichment of RRGs in transitional cells (Group1) (Figure 1I), we hypothesized that these cells might share reprogramming features with injury‐induced LPLCs [10]. To test this hypothesis, we integrated scRNA‐seq data derived from DDC diet‐induced mice at 1 week (GSE212692 [10], containing LPLCs). 3D analyses (3D‐PCA and 3D‐DC) revealed that DDC_1 W cells occupied a unique spatial position: diverging from both the PC3‐based normal maturation trajectory (black line) and the differentiation path of transitional cells (Tr_1W‐3 W; red line) (Figure S4C). Hierarchical clustering (Figure 2F) further identified four distinct groups: (1) immature hepatocytes (IH, P1‐P14), (2) mature hepatocytes (MH, P21‐Adult), (3) transplanted reprogrammed cells (Tr, Tr_1W‐3 W), and (4) DDC‐reprogrammed cells (DDC, DDC_1 W).

Gene set enrichment analysis (GSEA; Figure 2G) revealed that Tr cells exhibited closer transcriptional resemblance to IH cells, most notably through significant activation of the Cell cycle pathway. In contrast, DDC cells retained core hepatocyte maturity markers while acquiring cholangiocyte signature genes (e.g., *Sox9*, *Krt19*), confirming their bipotentiality. Critically, gene expression profiling coupled with immunofluorescence staining (Figure 2H,I; Figure S4D) demonstrated persistent hepatocyte identity (FAH^+^HNF4α^+^) and absence of cholangiocyte markers (*Sox9* ^−^CK19^−^) in tdTomato^+^AFP^+^ transplanted cells. Taken together with lineage tracing evidence, these results establish that although transitional cells share partial reprogramming features with LPLCs, they diverge fundamentally in differentiation trajectories and fate commitment. Given ubiquitous *Afp* expression in these cells, we formally designate them as *Afp* ^+^ reprogrammed hepatocytes (*Afp* ^+^ rHeps).

Next, we identified 78 genes enriched in *Afp* ^+^ rHeps, constituting the *Afp* ^+^ rHep‐related signature (ARS) (Materials and Methods, Table S7). Cell‐wise ARS module scoring (Figure 2J) robustly discriminated *Afp* ^+^ rHeps (Group1) from mature hepatocyte populations (Group2). Mirroring RRGs, ARS exhibited specific enrichment exclusively in reprogrammed hepatocytes (Tr and DDC) (Figure S4G). Pathway analysis (Figure 2K) demonstrated significant ARS gene involvement in Liver Regeneration, Lipid Metabolism (including PPAR signaling), and Detoxification Metabolism, aligning with their metabolic remodeling phenotype.

To assess whether the ARS signature reflects a model‐specific state or a generalizable feature of regenerating hepatocytes, we performed an integrated cross‐model analysis. We first identified transcriptionally distinct, regeneration‐active time points in multiple acute injury models (PHx, CCl_4_, APAP; Figure S4H). Using the ARS and RRG scores as quantitative proxies for unipotent and bipotent reprogramming states, respectively,​ we assessed these populations alongside LPLCs and biliary epithelial cells (BECs). The analysis revealed a clear stratification (Figure S4I, left). All hepatocyte populations, including *Afp* ^+^ rHeps, other injury‐induced hepatocytes, and adult hepatocytes, uniformly exhibited low RRG scores, distinguishing them from the high RRG scores of BECs and bipotent LPLCs. Within this hepatocyte lineage, *Afp* ^+^ rHeps were uniquely characterized by high ARS scores, marking them as a distinct, unipotent reprogramming state. Complementary lineage signature scoring confirmed the functional identity of each group (Figure S4I, right). Thus, while a low bipotent reprogramming signature (RRG score) is a shared feature of hepatocytes engaged in acute, parenchyma‐restoring repair, a high ARS score defines a specialized, optimized regenerative module that is most prominently activated in the *Afp* ^+^ rHep state.

### 2.3 *Afp* ^+^ rHeps Represent a Metabolically Optimized State through Coordinated Activation of a Complete Energy‐Production Axis

To define the metabolic state of *Afp* ^+^ rHeps, we performed gene set variation analysis (GSVA) based on KEGG metabolic pathways. This revealed their specific activation of Glycolysis, the TCA cycle, Oxidative Phosphorylation (OXPHOS), and amino acid metabolism pathways (Figure S4J), indicating a broad enhancement of energy metabolism. To determine whether these transcriptional changes translated into functional remodeling, we estimated single‐cell metabolic fluxes using scFEA [36]. *Afp* ^+^ rHeps exhibited significantly increased flux through key nodes, including pyruvate (Glycolysis end‐product), oxaloacetate (TCA cycle intermediate), and acetyl‐CoA (central metabolic hub) (Figure S4K), confirming the establishment of an active metabolic network primed for energy production and biomass synthesis.

Having established the intrinsic metabolic features of *Afp* ^+^ rHeps, we next sought to define the specificity of this state by positioning it within a broader regenerative landscape encompassing acute injury models (PHx, CCl_4_, APAP) and LPLCs. PCA of metabolic pathway enrichment scores revealed that transplanted *Afp* ^+^ rHeps (Tr_1 W, Tr_3 W) occupied a spatially distinct cluster (Quadrant I), separate from all other regenerating populations (Figure 2L). This apparent uniqueness, however, belied a shared functional context. Detailed pathway analysis showed that *Afp* ^+^ rHeps, LPLCs, and APAP‐regenerating hepatocytes all engaged in broad metabolic reprogramming, in contrast to the more restricted profiles of PHx and CCl_4_ models (Figure 2M). Thus, *Afp* ^+^ rHeps achieve a unique metabolic position precisely within this common landscape of extensive reprogramming. This finding prompted us to define the features that distinguish this state.

Within this shared landscape, we then asked what distinguished *Afp* ^+^ rHeps. Direct comparison of core energy‐production modules revealed that *Afp* ^+^ rHeps exhibited a markedly stronger enrichment of OXPHOS and the TCA cycle compared to other broadly activating states (Figure 2N; Figure S4L). Critically, the metabolic signature of *Afp* ^+^ rHeps was defined not by isolated pathways but by the sustained and coherent activation of an entire functional axis. A focused analysis showed that high *Afp* expression was concurrent with the coordinated upregulation of PPAR signaling (α/γ), fatty acid oxidation (FAO), the TCA cycle, and OXPHOS (Figure 2O). This PPAR‐FAO‐TCA‐OXPHOS cascade represents the complete, high‐fidelity execution of mitochondrial energy production. While a transient, partial activation of this axis was seen in APAP_48 h hepatocytes, only *Afp* ^+^ rHeps sustained both high *Afp* expression and high enrichment across its full length.

Thus, cross‐model analysis establishes *Afp* ^+^ rHeps as the archetype of a functionally optimized metabolic state, characterized by the coordinated PPAR‐FAO‐TCA‐OXPHOS energy‐production axis and co‐defined by sustained high *Afp* expression. This specialized program correlates with their capacity for massive liver repopulation.

### 2.4 Metabolism‐Proliferation Coupling Underlies *Afp* ^+^ rHep Heterogeneity

**FIGURE 3:**
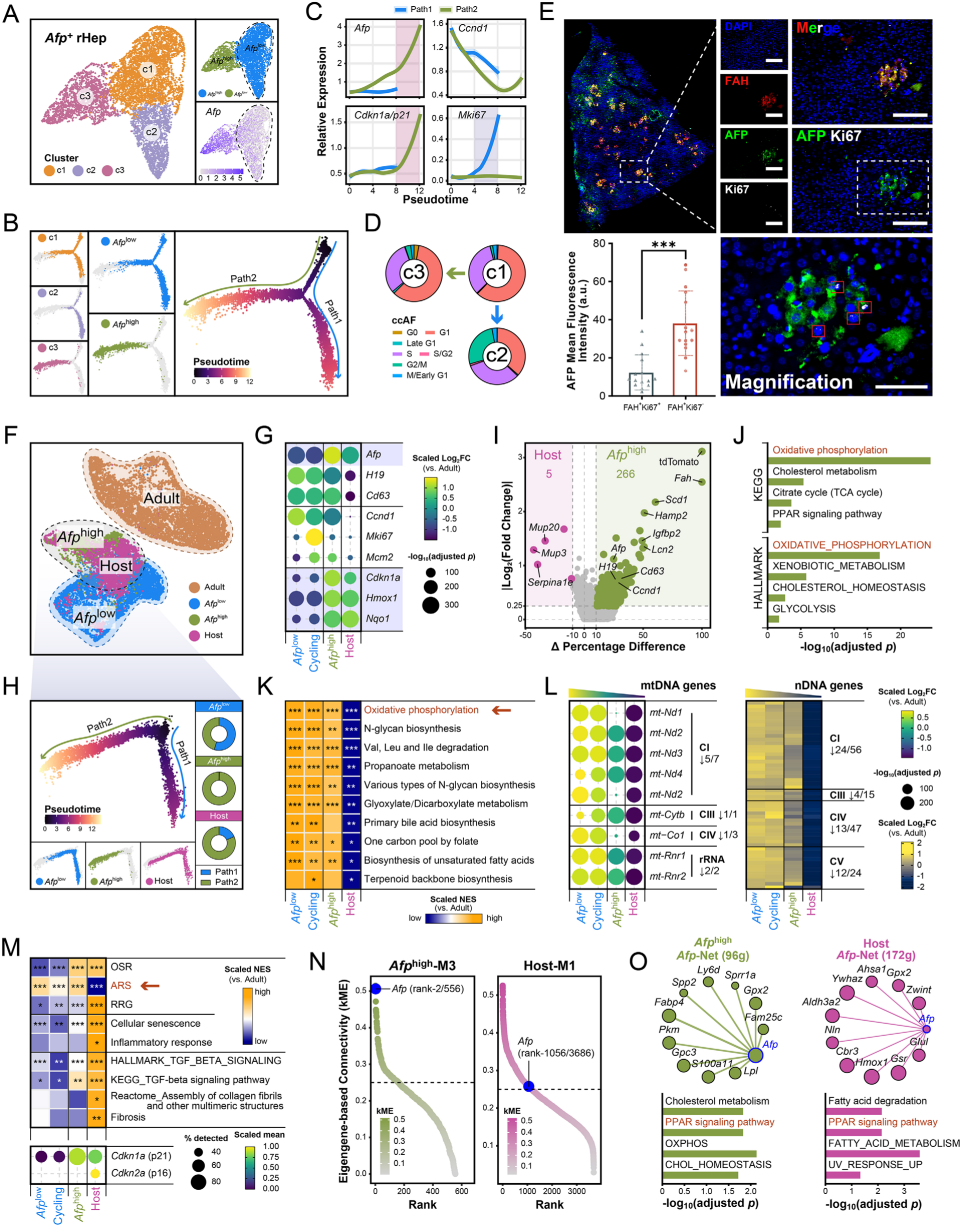
*Afp* marks a transitional hepatocyte state, enabling metabolic adaptation during liver injury. Additional details are provided in Table S3. (A) UMAP visualization of *Afp* ^+^ rHeps colored by cell clusters (**left**), cell subpopulations (**top right**), and *Afp* expression levels (**bottom right**). (B) Pseudotime analysis of *Afp* ^+^ rHeps was performed using Monocle2 [38], with cells colored by cell clusters (**left**), cell subpopulations (**middle**), and pseudotime (**right**). In the pseudotime plot, colored curves illustrate two distinct differentiation branches (Path1 and Path2). (C) Line plots illustrate the dynamic patterns of four genes along the pseudotime trajectory, with lines colored by distinct paths. The x‐axis denotes pseudotime during cell differentiation, and the y‐axis represents gene expression levels. Segment bandwidths indicate confidence intervals. (D) Donut charts show the ccAF [78]‐predicted cell cycle phase distributions across the three cell clusters. (E) Multiplex IF staining for AFP, Ki67, and FAH in host livers at 3 weeks post‐transplantation. Scale bars:100 µm. Quantification of AFP mean fluorescence intensity (MFI) in two transplanted hepatocyte subpopulations: FAH^+^Ki67^+^ (proliferative) and FAH^+^Ki67^−^ (non‐proliferative). ^***^ *p* < 0.001. (F) UMAP visualization of hepatocytes, colored by their classification into four types (Adult/Donor, *Afp* ^low^, *Afp* ^high^, and Host). (G) Dot plot showing the differential expression of feature genes across refined *Afp* ^+^ hepatocyte populations relative to Adult hepatocytes. The *Afp* ^low^ population from Figure 3F was subdivided into *Afp* ^low^ (non‐cycling) and *Afp* ^low^‐cycling subsets by removing cell cycle effects, and analyzed alongside *Afp* ^high^ and Host populations. Color intensity represents the scaled log_2_FC, while dot size corresponds to statistical significance (‐log_10_(adjusted *p*)). (H) Pseudotime analysis of *Afp* ^+^ hepatocytes (from Figure 3F) using Monocle2 reveals two distinct differentiation branches (Path1 and Path2). Cells are colored by pseudotime progression (**top left**) and subpopulation identity (**bottom left**). Donut charts (**right**) quantify path distribution frequencies across the three major populations (*Afp* ^low^, *Afp* ^high^, Host). (I) Volcano plot based on the percentage difference (x‐axis) and the |log_2_FC| (y‐axis) shows the differentially expressed genes (DEGs) in Host vs. *Afp* ^high^. Annotation highlights marker genes for each cell type. The counts of DEGs are annotated on the plot. (J) Bar plot showing the selected KEGG and HALLMARK pathways enriched for the upregulated genes in *Afp* ^high^ rHeps relative to Host hepatocytes (adjusted *p* < 0.05). The x‐axis represents the ‐log_10_(adjusted *p*) of the enriched pathways. (K) Heatmap showing the average NES of metabolic pathways for the *Afp* ^+^ hepatocyte populations relative to Adult hepatocytes. Asterisks mark significant enrichment (^*^adjusted *p* < 0.05, ^**^adjusted *p* < 0.01, ^***^adjusted *p* < 0.001). (L) Differential expression of electron transport chain (ETC) genes across *Afp* ^+^ hepatocyte populations relative to Adult hepatocytes. The dot plot (**left**) displays mitochondrial DNA (mtDNA)‐encoded ETC genes, and the heatmap (**right**) depicts nuclear DNA (nDNA)‐encoded ETC genes. Only genes with adjusted *p* < 0.05 and log_2_FC > 0.25 are shown. Color intensity represents the scaled log_2_FC, while dot size corresponds to statistical significance (‐log_10_(adjusted *p*)). (M) Heatmap showing the average NES of indicated pathways for the *Afp* ^+^ hepatocyte populations relative to Adult hepatocytes (**top**). Asterisks denote significant enrichment (^*^adjusted *p* < 0.05, ^**^adjusted *p* < 0.01, ^***^adjusted *p* < 0.001). Dot plot (**bottom**) showing the scaled expression (color intensity) and cellular percentage (dot size) of *Cdkn1a* and *Cdkn2a* across the populations. (N) Rank‐ordered gene plots based on module eigengene‐based connectivity (kME) values for the hdWGCNA [79]‐identified *Afp* ^high^‐M3 module (**left**, in *Afp* ^low^ rHeps) and the Host‐M1 module (**right**, in Host hepatocytes). The *Afp* gene position in each ranking is indicated by blue dots. (O) Network diagrams of *Afp*‐first‐order interactors within the hdWGCNA‐identified *Afp* ^high^‐M3 (**top left**) and Host‐M1 (**top right**) modules, showing the top 10 genes ranked by kME. Node size is scaled by kME value, and edge thickness is weighted by connection strength, representing co‐expression relationships. The *Afp* gene is colored blue. Bar plots (**bottom**) show the selected KEGG and HALLMARK pathways significantly enriched (adjusted *p* < 0.05) for the genes from each respective submodule. The x‐axis indicates the ‐log_10_(adjusted *p*) of the enrichment analysis.

Pseudotime analysis using Monocle 3 [37] revealed a branched trajectory in *Afp* ^+^ rHeps (Figure S5A), indicating intrinsic cellular heterogeneity. From the clustering results, we further classified three clusters (c1−c3) into two distinct subpopulations based on *Afp* expression levels: *Afp* ^low^ (c1−c2) and *Afp* ^high^ (c3) (Figure 3A). To resolve their differentiation relationships, we integrated four trajectory analysis approaches: first constructing cellular developmental trajectories using Monocle 2 [38] (Figure 3B), followed by incorporating Monocle 3‐calculated pseudotime, scVelo [39]‐inferred RNA velocity, and Slingshot [40]‐reconstructed lineages (Figure S5B). This consensus analysis identified two independent paths: Path1 captured intra‐*Afp* ^low^ differentiation (c1→c2), while Path2 represented a fate transition from *Afp* ^low^−c1 to *Afp* ^high^−c3 cells.

GO and trend analyses of pseudotime gene modules (Figure S5C) uncovered divergent functional specialization among *Afp* ^+^ rHep subpopulations: *Afp* ^low^‐c1 cells are primarily engaged in substance metabolism; *Afp* ^low^−c2 cells show significant enrichment of cell cycle‐related genes, displaying characteristics of cycling cells; while *Afp* ^high^−c3 cells specifically activated Xenobiotic stimulus signaling concomitant with the expression of the cell cycle arrest marker *Cdkn1a*/p21 (Figure 3C). Differential expression analysis of *Afp* ^low^ vs. *Afp* ^high^ groups (Figure S5D) revealed that *Afp* ^high^ cells upregulated FAs transport genes (*Fabp4*) and antioxidant genes (*Hmox1*, *Nqo1*), whereas *Afp* ^low^ cells preferentially expressed genes governing FA desaturation (*Scd1*), glucose transport (*Slc2a2*), and cell cycle (*Ccnd1*) progression. Pathway analysis (Figure S5E) highlighted their metabolic complementarity: *Afp* ^high^ cells dominated xenobiotic metabolism and FA β‐oxidation, while *Afp* ^low^ cells specialized in energy production and cholesterol biosynthesis.

Metabolic analysis uncovered distinct subpopulation specialization (Figure S5F,G): *Afp* ^low^ cells exhibited proliferative metabolism with enhanced Glycolysis, TCA cycle, and OXPHOS. Conversely, *Afp* ^high^ cells displayed reduced mitochondrial flux but upregulated most FA β‐oxidation genes (spanning both mitochondrial and peroxisomal pathways) while preserving basal glycolytic activity. This metabolic reprogramming critically enhances *Afp* ^high^ cell fitness in the stressed hepatic niche. Crucially, the two subpopulations orchestrate liver regeneration through complementary metabolic division of labor: *Afp* ^low^ cells supply energy and membrane biosynthesis precursors, while *Afp* ^high^ cells specialize in lipid catabolism and xenobiotic detoxification.

In addition, cell cycle analysis (Figure 3D; Figure S5B) demonstrated that Path1‐terminal c2 cells (*Afp* ^low^‐cycling) exhibited proliferative traits with increased G2/M phase proportion. Conversely, Path2‐terminal c3 cells (*Afp* ^high^) maintained a cell cycle distribution comparable to early‐stage c1 cells (*Afp* ^low^), despite occupying a pseudotime‐terminal position. Molecular profiling revealed that *Afp* ^high^ cells highly expressed the cell cycle inhibitor *Cdkn1a*/p21 (Figure 3C). At the protein level, AFP mean fluorescence intensity was notably low specifically within the proliferative (FAH^+^Ki67^+^) compartment (Figure 3E). The observation that proliferating cells harbor low AFP protein is consistent with the inverse relationship defining *Afp* ^high^ cells (high AFP, low proliferation) and further substantiates their proliferation‐restricted state. Taken together, these data indicate that *Afp* ^high^ cells maintain viability and functionality in the injured microenvironment through a coupled “metabolic adaptation‐proliferation arrest” phenotype.

### 2.5 Divergent Metabolic Adaptations of *Afp* ^+^ Hepatocytes in Injured Liver are Dictated by the Topological Position of *Afp* within Co‐Expression Networks

To investigate potential interactions between *Afp* ^+^ rHeps and host hepatocytes in the injury‐activated niche, we characterized *Fah* ^−/−^ host hepatocytes. Integrated analysis showed that host hepatocytes (Host) co‐clustered with *Afp* ^+^ rHeps (*Afp* ^low^/*Afp* ^high^) while being distinct from mature hepatocytes (Adult) (Figure 3F). Further comparative analysis revealed consistent traits between Host cells and *Afp* ^high^ cells: (1) Comparable expression levels of *Afp* (key marker gene), *Cdkn1a* (cell cycle arrest‐related gene), *Hmox1*, and *Nqo1* (antioxidant‐related genes) (Figure 3G); (2) Similar cell cycle distribution patterns (Figure S5H); (3) Predominant localization in the Path2 branch (*Afp* ^low^→*Afp* ^high^ direction) within the Monocle 2‐reconstructed developmental trajectory (Figure 3H). Collectively, these data demonstrate that *Fah* ^−/−^ *Afp* ^+^ host hepatocytes acquire *Afp* ^high^‐like proliferation‐arrested properties under injury conditions.

However, in‐depth analysis revealed fundamental differences between Host and *Afp* ^high^ cells at the molecular regulatory level. Compared to *Afp* ^high^ cells, Host cells exhibited marked transcriptional dysregulation, with only 5 genes upregulated vs. 266 downregulated (Figure 3I). These included *Afp* ^+^ rHep signature genes (*Afp*, *H19*, *Cd63*, *Ccnd1*) and Zone2 hepatocyte markers (*Hamp2*, *Igfbp2*) [41, 42, 43]. The downregulated genes were significantly enriched in metabolic pathways, particularly OXPHOS (Figure 3J; Figure S5I,J). GSEA of KEGG metabolic pathways (with proliferative *Afp* ^low^−c2 cells separately grouped as ‘Cycling’ to control for cell cycle effects) revealed specific suppression of OXPHOS in Host cells (Figure 3K), indicating impaired ATP synthesis capacity. Broad suppression of genes encoding electron transport chain (ETC) components (mtDNA‐ and nDNA‐derived) was observed in Host cells (Figure 3L; Figure S5K), indicative of profound mitochondrial impairment. Notably, Host cells also displayed defects in DNA repair pathways (e.g., Nucleotide excision repair, Double‐strand break repair) and mitochondrial quality control mechanisms (e.g., Autophagy, Mitophagy), indicating compromised genomic stability maintenance (Figure S5L).

We next identified a pronounced expression gradient of *Afp* across the three *Afp* ^+^ populations (*Afp* ^high^ > Host > *Afp* ^low^), which strongly correlated with PPAR signaling activity levels (Figure 3G,J; Figure S5E,J). Correlation analysis confirmed a strong positive association between PPAR signaling and *Afp* expression levels (Spearman's *ρ* = 0.73; Figure S5M). Strikingly, Host cells showed markedly reduced ARS enrichment (Figure 3M) despite detectable PPAR signaling, contrasting with the robust association between the signature and PPAR pathway.

Co‐expression network analysis identified *Afp*‐containing gene modules in both *Afp* ^high^ (*Afp* ^high^‐M3) and Host (Host‐M1) cells, though their regulatory hierarchies differed significantly. In *Afp* ^high^ cells, *Afp* functioned as a central regulatory hub (ranked second, kME = 0.51) within a tightly organized network of 556 genes. Conversely, in Host cells, *Afp* showed significantly reduced regulatory prominence (ranked 1056th, kME = 0.26) and was embedded in a more diffuse network architecture encompassing 3,686 genes (Figure 3N). Concomitantly, Host cells displayed: (1) activation of RRGs [10], (2) enrichment of cellular senescence markers (e.g., *Cdkn1a*/p21, *Cdkn2a*/p16), and (3) enhanced inflammatory responses coupled with extracellular matrix (ECM) deposition (Figure 3M). Functional enrichment analysis revealed that despite structural differences in their networks, the *Afp*‐associated subnetworks in both cell types maintained high functional consistency in PPAR‐centered lipid metabolism regulation (Figure 3O). These results demonstrate conserved core functionality in *Afp*‐related gene modules across both *Afp* ^+^ rHeps and *Afp* ^+^ host hepatocytes, where functional divergence arises principally from the distinct positioning of *Afp* within these networks.

### 2.6 AFP Drives Liver Repair through a PPARγ‐Centered Transcriptional‐Metabolic Network

**FIGURE 4:**
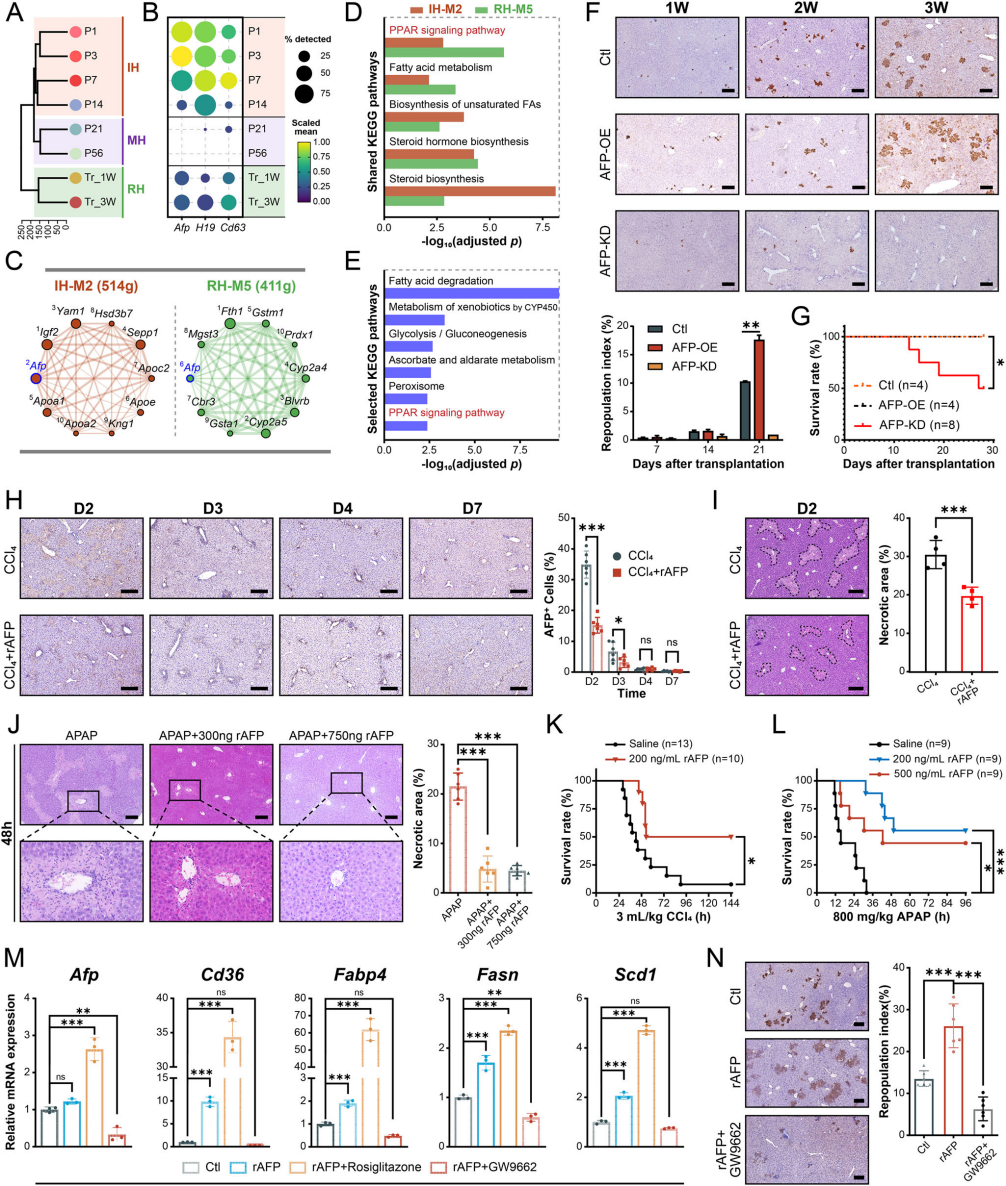
AFP drives liver repair through PPAR‐centric transcriptional and metabolic networks in liver injury. Additional details are provided in Table S4. (A) Hierarchical clustering based on transcriptomic profiles distinguishes three distinct populations (IH, MH, RH) among postnatal (P1‐P56) and transplanted (Tr_1W‐Tr_3 W) hepatocytes. (B) Dot plot showing the scaled expression (color intensity) and cellular percentage (dot size) of *Afp*, *H19*, and *Cd63* across postnatal (P1‐P56) and transplanted hepatocytes (Tr_1W‐Tr_3 W). (C) Network diagrams of the top 10 genes (ranked by kME) within the hdWGCNA‐identified IH‐M2 module (**top**, in IH group) and the RH‐M5 module (**bottom**, in RH group). Node size is scaled by kME value, and edge thickness is weighted by connection strength, representing co‐expression relationships. The *Afp* gene is highlighted in blue. (D) Bar plot showing significantly enriched KEGG pathways shared between the *Afp*‐associated gene modules IH‐M2 and RH‐M5 (adjusted *p* < 0.05). A dashed box denotes lipid metabolism‐related signaling. The x‐axis indicates the ‐log_10_(adjusted *p*) of the enrichment analysis. (E) KEGG Pathways significantly enriched (adjusted *p* < 0.05) among the potential AFP‐interacting genes identified in both Ctl and AFP‐OE samples (listed in Table S4). The x‐axis represents the ‐log_10_(adjusted *p*‐value) for each pathway. (F) (**Top**) Representative IHC staining for FAH (a marker of donor repopulation) in liver sections of *Fah* ^−/−^ host mice with varying AFP backgrounds (Ctl, AFP‐OE, AFP‐KD) at 1, 2, and 3 weeks post‐transplantation with normal adult tdTomato^+^ hepatocytes. Scale bars: 100 µm. (**Bottom**)​ Quantification of the repopulation index, presented as the percentage of donor‐derived (tdTomato^+^) area, in the three recipient groups over time. ^**^ *p* < 0.01. (G) Kaplan‐Meier survival analysis of *Fah* ^−/−^ mice following hepatocyte transplantation, stratified by their systemic AFP background (Ctl, n = 4; AFP‐OE, n = 4; AFP‐KD, n = 8). ^*^ *p* < 0.05 (AFP‐KD vs. Ctl/AFP‐OE). (H) (**Left**) Representative IHC staining of AFP in liver sections. Mice were treated with CCl_4_ alone or CCl_4_ combined with rAFP injection, and tissues were harvested at 2, 3, 4, and 7 days post‑administration. Scale bars: 100 µm. (**Right**) Quantification of AFP^+^ cells, presented as the percentage of AFP^+^ cells per field, in the two treatment groups across the indicated timepoints. ns: not significant, ^*^ *p* < 0.05, ^***^ *p* < 0.001. (I) Representative H&E staining and quantification of necrotic area in liver sections from mice at 2 days postadministration. Mice were treated with CCl_4_ alone or CCl_4_ combined with rAFP injection. Scale bars: 100 µm. ^***^ *p* < 0.001. (J) Representative H&E‐stained liver sections from mice 48 h after intraperitoneal injection of 300 mg/kg APAP. Mice were concurrently treated with either vehicle (Control), low‐dose rAFP, or high‐dose rAFP. Necrosis is markedly reduced in rAFP‐treated livers (^***^ *p* < 0.001). Scale bars: 100 µm. (K) Kaplan‐Meier survival curves following a single intraperitoneal injection of a lethal dose of CCl_4_ (3 mL/kg). Mice were treated with either CCl_4_ alone (Control) or CCl_4_ followed by rAFP treatment. ^*^ *p* < 0.05. (L) Kaplan‐Meier survival curves of mice following a lethal dose of APAP (800 mg/kg) with or without rAFP treatment (based on the circulating blood volume of mice). ^*^ *p* < 0.05, ^***^ *p* < 0.001. (M) Quantitative RT‐PCR (qRT‐PCR) analysis of gene expression in primary mouse hepatocytes treated for 24 h with: control, rAFP alone, rAFP combined with the PPARγ agonist Rosiglitazone (10 µm), or rAFP combined with the PPARγ antagonist GW9662 (10 µm). mRNA levels were assessed for the *Afp* gene and four canonical downstream genes of the PPARγ signaling pathway (*Cd36*, *Fabp4*, *Fasn*, *Scd1*). ^*^ *p* < 0.05, ^**^ *p* < 0.01, ^***^ *p* < 0.001. (N) (**Left**) Representative FAH IHC staining of liver sections from *Fah* ^−/−^ mice at 3 weeks post‐transplantation. Mice were treated as indicated: Basal, rAFP, or rAFP+GW9662. Scale bars: 200 µm. (**Right**) Quantification of the repopulation index, presented as the percentage of donor‐derived (FAH^+^) area, in the three treatment groups. ^***^ *p* < 0.001.

To define the transcriptional network associated with *Afp* in regenerating hepatocytes, we integrated single‑cell data from transplanted *Afp* ^+^ rHeps and postnatal hepatocytes. Unsupervised clustering distinguished immature (IH), mature (MH), and reprogrammed hepatocyte (RH) states (Figure 4A). RH cells, like IH cells, expressed high levels of *Afp*, *H19*, and *Cd63* (Figure 4B) and produced predominantly non‑fucosylated AFP (Figure S6A). Weighted gene co‑expression network analysis identified *Afp* as a central hub within core modules in both IH and RH cells (Figure 4C), with both modules significantly enriched for PPAR‑centered lipid metabolism pathways (Figure 4D). This indicates conservation of AFP‑associated regulatory logic between development and regeneration.

We next delineated the protein interactome of intracellular AFP in transplanted hepatocytes through coimmunoprecipitation coupled with mass spectrometry (Co‐IP‐MS), comparing cells from control (Ctl) vs. *Afp‐*overexpressing (AFP‐OE) host livers. Pathway analysis of the shared interactors confirmed enrichment for PPAR signaling and lipid metabolism (Figure 4E), establishing that intracellular AFP operates within this metabolic regulatory network.

To test the functional role of AFP in liver repair, we modulated its expression in the *Fah* ^−/−^ transplantation model. Efficient knockdown (AFP‑KD) or overexpression (AFP‑OE) was confirmed by immunohistochemistry (IHC) and western blot (WB) (Figure S6B,C). AFP‑OE significantly enhanced donor hepatocyte repopulation and host survival, whereas AFP‑KD severely impaired repopulation and led to uniform mortality (Figure 4F,G; Figure S6B). This demonstrates that host‑derived AFP is both necessary and sufficient for successful liver repopulation.

To establish the broader relevance of AFP across acute injury, we extended our analysis to CCl_4_ and APAP models. *Afp* expression was rapidly induced in host hepatocytes after injury (Figure S6D,E,L). More importantly, therapeutic administration of recombinant AFP (rAFP) conferred potent hepatoprotection. In CCl_4_‑injured mice, rAFP reduced tissue necrosis (Figure 4H,I) and significantly improved survival following a lethal dose (Figure 4K). Similarly, in APAP‑induced injury, rAFP markedly attenuated hepatocellular necrosis (Figure 4J; Figure S6F) and rescued mice from lethal liver failure (Figure 4L). These results demonstrate that AFP‑mediated protection is a generalizable mechanism operative across distinct etiologies of acute liver injury.

To mechanistically link AFP to PPARγ signaling, we first identified PPARγ as a putative transcriptional regulator of *Afp* through integrated motif and regulon analysis (Figure S6,G‑K). We then performed direct perturbation experiments. In vitro, treatment of primary hepatocytes with rAFP activated expression of key PPARγ‐regulated genes involved in lipid metabolism (*Cd36*, *Fabp4*, *Fasn*, *Scd1*). Moreover, rAFP‐induced *Afp* expression itself was further enhanced by a PPARγ agonist and suppressed by a PPARγ antagonist (Figure 4M). Together, these results place AFP within a PPARγ‐dependent positive feedback loop, wherein AFP activates PPARγ signaling, which in turn sustains *Afp* expression. This loop is functionally essential, as the prorepopulation effect of rAFP in vivo was entirely abolished by coadministration of the PPARγ antagonist GW9662 (Figure 4N).

To identify the core transcriptional program co‑activated with *Afp* during regeneration, we analyzed hepatocytes from three distinct injury models (PHx, CCl_4_, and APAP).​ Regenerating hepatocytes consistently showed co‑enrichment of three key signature modules: the ARS (*Afp* ^+^ rHep‑related), the RH‑M5 (*Afp*‑correlated), and a focused PPARγ signature [44] (Figure S6M). This multi‑signature overlap indicates that the AFP‑associated (and PPARγ‑centered) regenerative program, whose core signatures were defined from donor‑derived Afp^+^ rHeps repopulating the *Fah* ^−/−^ liver, is a conserved feature of acute liver repair across diverse mouse injury etiologies.

Having established the conservation of this program in mouse acute injury, we asked whether it is active in human liver regeneration.​ In a public single‑cell dataset from patients with APAP‐induced acute liver failure (APAP‐ALF) and non‐A‐E hepatitis ALF (NAE‐ALF) [45], regenerating hepatocytes showed significant co‑enrichment of the same three key signatures: the ARS, the RH‑M5 module, and the PPARγ signature (Figure S6N). While this cross‑species comparison involves distinct initiating etiologies, the conserved activation of this core transcriptional module supports its fundamental role in acute repair. This conservation underscores the translational relevance of the PPARγ/AFP‑driven regenerative program.

Collectively, these data establish AFP as a central effector of liver repair that functions through a self‑reinforcing, positive feedback loop that amplifies PPARγ signaling and its own expression. This mechanism is necessary for its pro‑repair effects, conserved from mouse models to human liver failure, and underpins its therapeutic potential.

### 2.7 Neutrophil‐Derived TNF‐α Promotes the Proliferation of Transplanted Hepatocytes by Activating AP‐1

Although AFP is indispensable for cell survival and crucial for regeneration of damaged livers, we found that the highly proliferative *Afp* ^low^ subset paradoxically exhibits minimal AFP expression (Figure 3A,G). This prompted us to hypothesize that an AFP‐independent mechanism drives hepatocyte proliferation. Regulatory network analysis nominated a module of six AP‐1 TFs (JUN, JUNB, JUND, FOS, FOSB, ATF3; termed “6A”) as core regulons in *Afp* ^+^ rHeps (Figure S7A), consistent with their broad upregulation (Figure S7B). Chromatin accessibility profiling confirmed significant enrichment of AP‐1 binding motifs in *Afp* ^+^ rHeps, particularly the *Afp* ^low^ subset (Figure S7C–E).

**FIGURE 5:**
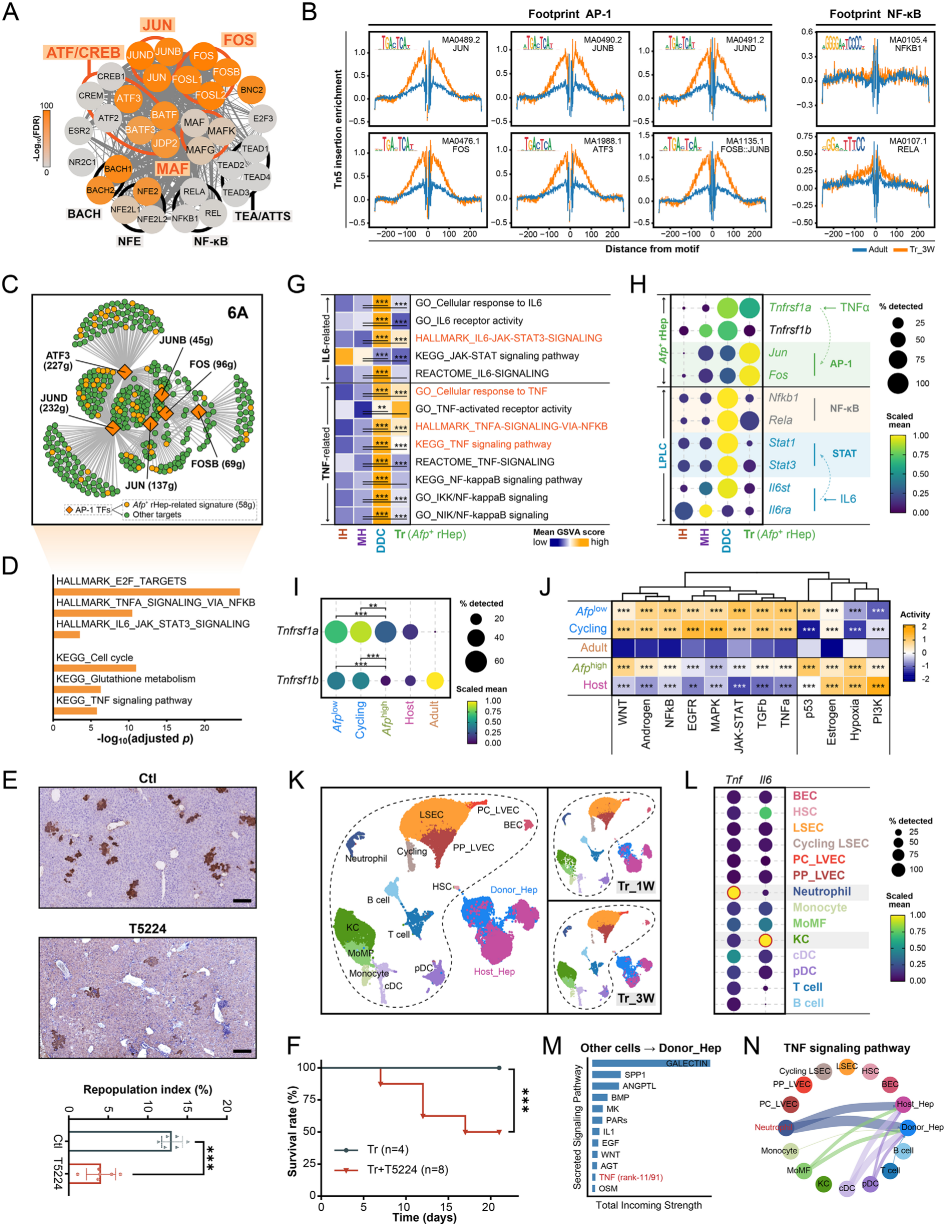
TNF‐α stimulates reprogrammed hepatocyte proliferation by activating AP‐1. Additional details are provided in Table S5. (A) Protein‐protein interaction network of TFs with enriched binding motifs (false discovery rate, FDR < 0.05) in Tr_3 W cells. Interactions were inferred by StringDB, with edge thickness proportional to the combined score. Node colors indicate ‐log_10_(FDR) of the motif enrichment analysis. (B) Tn5 insertion enrichment profiles around the center of AP‐1 (n = 6 motifs) and NF‐κB (n = 2 motifs) motifs in Tr_3 W hepatocytes (orange) and Adult hepatocytes (blue). The x‐axis represents the distance from the motif center. The y‐axis shows the normalized Tn5 insertion frequency. (C) Gene regulatory network inferred from pySCENIC [80], showing the transcriptional regulatory relationships between a module of six AP‐1 TFs (labeled as "6A") and their predicted target genes in *Afp* ^+^ rHeps. Fifty‐eight of these target genes overlap with the ARS. (D) Bar plot showing the selected HALLMARK and KEGG pathways that are significantly enriched for 6A‐regulated target genes (adjusted *p* < 0.05). The x‐axis indicates the ‐log_10_(adjusted *p*) of the enriched pathways. (E) Representative FAH IHC staining and quantification of repopulation index in livers of surviving mice at 3 weeks post‐transplantation, with or without AP‐1 inhibitor (T5524) treatment. Scale bars: 200 µm. ^***^ *p* < 0.001. (F) Kaplan‐Meier survival curves of *Fah* ^−/−^ mice following hepatocyte transplantation with or without AP‐1 inhibitor (T5524) treatment. AP‐1 inhibitor treatment significantly reduced survival compared to the control group. ^***^ *p* < 0.001. (G) Average GSVA enrichment scores for IL6‐ and TNF‐related pathways in the groups defined in Figure 2F. Asterisks denote pathways that are significantly different in either the Tr or DDC group relative to the MH group (two‐sided Wilcoxon rank sum test, ^***^adjusted *p* < 0.001). (H) Dot plot showing the scaled expression (color intensity) and cellular percentage (dot size) of IL6‐ and TNF‐related genes across all groups in Figure 2F. (I) Expression of TNF‐α receptor genes. Dot plot showing the scaled expression (color intensity) and cellular percentage (dot size) of *Tnfrsf1a* (TNFR1) and *Tnfrsf1b* (TNFR2) across all cell groups defined in Figure 3F. (J) Clustered heatmap of 14 signaling pathway activities (inferred by PROGENy [47]) across the same cell groups as in Figure 3F. Both the *Afp* ^low^ and Cycling populations exhibit enhanced mitogenic signaling pathway activity. (K) UMAP visualization of integrated scRNA‐seq data from liver cells at 1 and 3 weeks post‐transplantation, showing integrated (**top**, combined) views and time‐resolved views (**bottom**, 1 and 3 W separately). Cells are colored by annotated cell type. Dotted lines demarcate non‐hepatocyte populations. (L) Dot plot showing the scaled expression (color intensity) and cellular percentage (dot size) of *Tnf* and *Il6* across non‐hepatocyte cell types. (M) Bar plot displaying the total incoming communication strength of ligand‐receptor interactions for each secretory signaling pathway targeting Donor_Hep. Pathways are ranked by their aggregate strength. (N) Circle plot depicting the inferred interaction strength of the TNF signaling pathway from non‐hepatocyte sender cells to hepatocyte receivers (Donor_Hep and Host_Hep).

This was reflected in potentiated AP‐1 TF activity (Figure 5A,B) and elevated integrated regulon activity (Figure 5B). The predicted targets of the 6A regulon encompassed most ARS genes (58 of 78; Figure 5C) and were enriched in Cell cycle and E2F_TARGETS (Figure 5D), directly linking 6A to proliferation. Accordingly, hepatocytes expanded ex vivo with TNF‐α exhibited enhanced 6A gene expression, regulon activity, and enrichment of ARS and 6A signatures (Figure S7G,H). The potency of this AP‐1‐driven program is further underscored by the highly synchronized activation of an AP‐1‐dependent regeneration response program (RRP) [46] in transplanted hepatocytes, a pattern distinct from the more variable activation seen during endogenous liver repair (Figure S7F).

To directly test the functional necessity of AP‐1 activity, we treated recipient mice with the AP‐1 inhibitor T‐5224 in the transplantation model. Pharmacological AP‐1 inhibition severely impaired donor hepatocyte repopulation (Figure 5E) and significantly reduced host survival (Figure 5F), establishing that AP‐1 activity is essential for successful liver repopulation.

We next sought the upstream signal activating AP‐1. The 6A regulon targeted inflammatory pathways mediated by TNF‐α and IL‐6 (Figure 5D). Pathway activity analysis revealed that *Afp* ^+^ rHeps mounted a selective response to TNF‐α, but not IL‐6, signaling (Figure 5G). This was underpinned by specific upregulation of the TNF‐α receptor gene *Tnfrsf1a* (TNFR1) in these cells (Figure 5H,I). PROGENy [47] analysis further confirmed enhanced mitogenic pathway activity, including TNF signaling, in the proliferative *Afp* ^low^ subsets (Figure 5J).

To identify the source of TNF‐α, we profiled the liver niche during repopulation (Figure 5K). Neutrophils emerged as the predominant expressers of *Tnf*, whereas kupffer cells (KCs) were the main source of *Il6* (Figure 5L). Cell‐cell communication analysis ranked TNF signaling among the top incoming pathways to donor hepatocytes (Figure 5M), primarily originating from neutrophils (Figure 5N; Figure S7J). The most robust interaction was between neutrophil‐derived TNF and donor hepatocyte‐expressed TNFR1 (Figure S7L). Neutrophils were also the key source of oncostatin M (OSM), another critical niche signal [48] (Figure S7J,K).

Collectively, these results define a neutrophil‐hepatocyte axis wherein neutrophil‐derived TNF‐α, signaling through TNFR1, activates the AP‐1 transcriptional module to license the proliferative entry of transplanted hepatocytes, while co‐delivered OSM likely safeguards their epithelial identity.

### 2.8 Transplanted Hepatocytes Exhibit Molecular Zonation Delays but Enhance Liver Repopulation via EMP‐Boosted Migration

The spatial zonation of hepatocytes along the portocentral axis establishes the fundamental basis for the compartmentalized execution of liver functions [49, 50, 51]. To define the zonation dynamics of repopulating donor hepatocytes, we calculated zonation scores using a published method [17] with partial least squares regression (PLSR) [52] based on 18 established zonation markers [41] (Figure S8A). These scores displayed maximal heterogeneity at the R0_0 W time point, sharply declined following transplantation, and subsequently underwent a steady increase during regeneration. This dynamic trajectory indicates an initial loss of native zonation, followed by a period of phenotypic imbalance, progressing toward the acquisition of new zonation properties within the regenerative niche. To decipher these evolving molecular features, we developed a high‐performance computational classifier for hepatocyte zonation (Materials and Methods). Validation on R0_0 W hepatocytes confirmed model reliability, as predictions recapitulated expected Zone1‐3 proportions (Figure S8C,H). Application of this classifier to host hepatocytes revealed altered zonation proportions (Figure S8I,J). Critically, analysis of donor *Afp* ^+^ subsets uncovered a divergence between their periportal location and acquired molecular identity. *Afp* ^high^ cells, despite being portal proximal, acquired a Zone 3like (central vein) transcriptional state, a profile consistent with their specialized lipid catabolism and detoxification functions and indicative of metabolically driven identity adaptation (Figure S8I).

**FIGURE 6:**
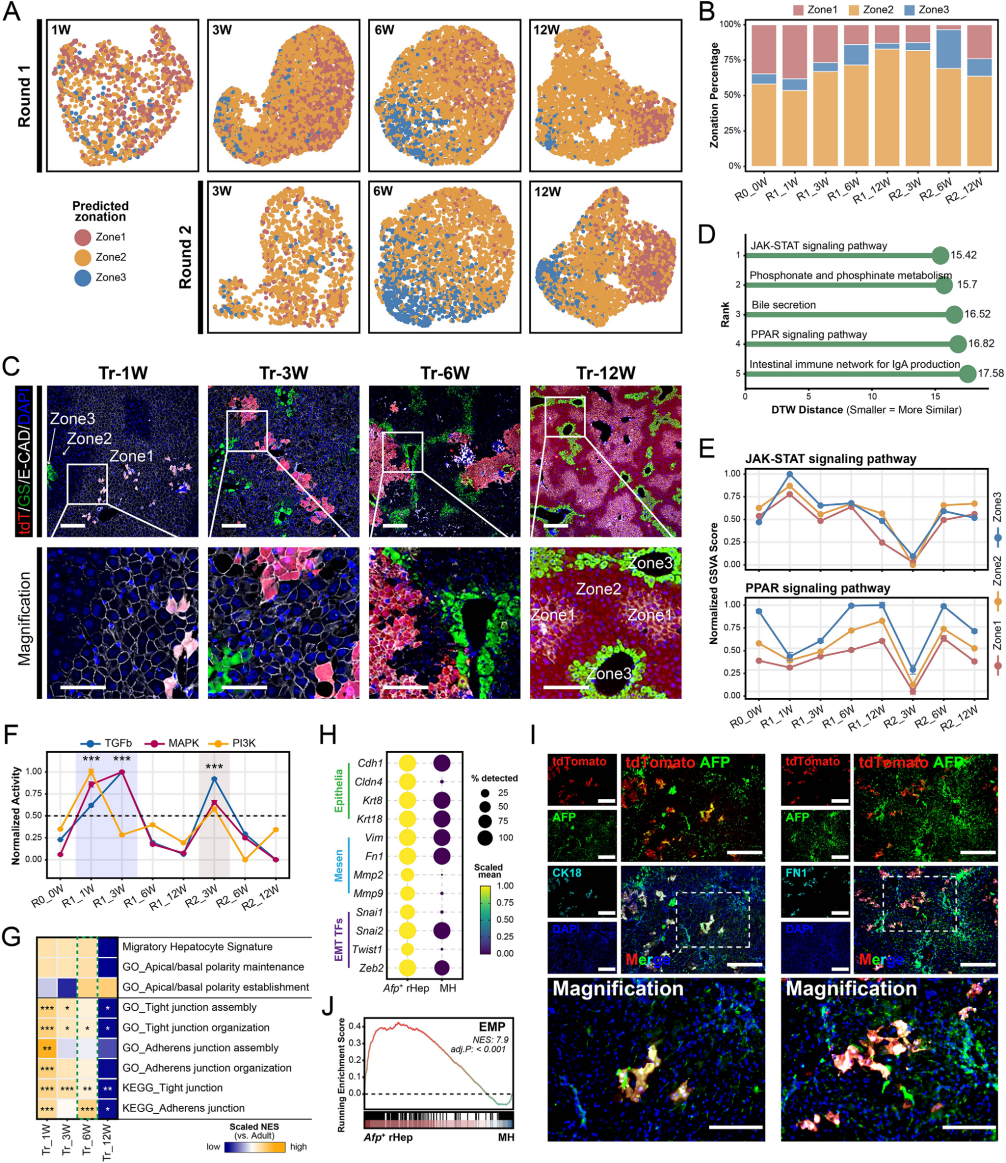
Zonation reconstruction of the host liver by the transplanted mature hepatocytes. Additional details are provided in Table S6. (A) UMAP visualization of transplanted hepatocytes from two rounds, collected at 1, 3, 6, and 12 weeks post‐transplantation, colored by model‐predicted cell zonation (Zone 1–3). (B) Stacked bar plot showing the distribution of predicted hepatocyte zonation (Zone1‐3) across eight samples. (C) IF staining for tdTomato, GS, and E‐CAD in livers at 1, 3, 6, and 12 weeks post‐transplantation (**top**). Zoom in on the selected field of view (**bottom**). Scale bars: 100 µm. (D) Lollipop plot of top 5 KEGG pathways most closely matched to hepatocyte zonation dynamics (across eight samples) as quantified by Dynamic Time Warping (DTW) [53] distance. Smaller DTW values indicate stronger alignment between pathway activity and zonation pattern changes. (E) Line plots illustrate the temporal dynamics of JAK‐STAT (**top**) and PPAR (**bottom**) pathway activities (GSVA scores) across hepatocyte zonation (Zone1‐3). Data points represent the mean scores of zonated hepatocytes within each group (stratified by zone and timepoint), and error bars denote the mean ± SEM. (F) Line plot showing the temporal dynamics of TGFb, MAPK, and PI3K pathway activities (inferred by PROGENy [47]) across eight samples. Data points represent the mean activity of hepatocytes in each sample, and error bars denote the mean ± SEM. Asterisks denote significantly upregulated pathways in each comparison (R1_1 W/3 W vs. R0_0 W and R2_3 W vs. R1_12 W; two‐sided Wilcoxon rank sum test, ^***^adjusted *p* < 0.001). (G) Heatmap showing the average NES of pathways related to cell junction and cell polarity for hepatocytes at each transplant timepoint relative to Adult hepatocytes. Asterisks mark significant enrichment (^*^adjusted *p* < 0.05, ^**^adjusted *p* < 0.01, ^***^adjusted *p* < 0.001). (H) Dot plot showing the scaled expression (color intensity) and cellular percentage (dot size) of epithelial mesenchymal transition (EMT)‐related genes in *Afp* ^+^ rHep and MH. (I) IF staining for tdTomato, AFP, and CK18 (**left**) or FN1 (**right**) in livers at 3 weeks post‐transplantation. Scale bars: 100 µm. (J) GSEA of epithelial mesenchymal plasticity (EMP) signature [54] in *Afp* ^+^ rHep vs. MH.

Reconstruction of molecular zonation across transplantation time points uncovered pronounced dynamic shifts in the spatial identity of donor hepatocytes within the host liver environment (Figure 6A,B). Specifically, Zone2 proportions exhibited a continuous expansion throughout the first‐round (R1) transplantation timeline. This trend was reversed in the second‐round (R2) paradigm, where Zone2 representation progressively contracted. Notably, Zone2 proportions converged to nearly identical values in R1_12 W and R2_3 W samples, concurrently reaching their peak levels within respective transplantation timelines. By the R2_12 W endpoint, Zone2 representation had rebounded to a level that closely approximated the native physiological baseline (R0_0 W).

We next examined the spatial positions of transplanted hepatocytes across four repopulation stages (Figure 6C; Figure S8J). At 1 week post‐transplantation, the host liver architecture remained intact, with donor cells exclusively colonizing Zone1. By week 3, architectural disruption became evident: the definitive GS^−^E‐CAD^−^ Zone2 niche was obliterated, in contrast to the expanding proportion of Zone 2‐assigned cells in molecular profiling (Figure S8I), coinciding with a wave of donor hepatocyte proliferation and initial invasion into adjacent zones. At week 6, near‐total host hepatocyte depletion was observed, enabling donor cell migration into Zone3 territories. By week 12, donor hepatocytes achieved complete liver repopulation, occupying all spatial domains of the hepatic lobule. These findings demonstrate that anatomical positioning stabilizes prior to molecular zonation maturity, establishing a sequential pattern in regenerating livers where spatial occupancy precedes functional specialization.

To delineate signaling pathways governing molecular zonation, we first quantified KEGG pathway activity in individual hepatocytes using gene signature scoring. Then, we employed dynamic time warping (DTW) [53] analysis to measure the temporal coupling between pathway activation dynamics and Zone1‐3 proportion fluctuations—where smaller DTW distances indicate higher trajectory similarity—and ranked all pathways by their association strength.This analysis nominated inflammatory and metabolic signaling pathways as the top five candidates (Figure 6D). Focused investigation of JAK‐STAT and PPAR signaling dynamics across zones and timepoints (Figure 6E) revealed consistent discrimination of hepatocyte identities across all three zones by PPAR activation patterns throughout the entire regeneration timeline. This suggests a fundamental role for PPAR in establishing and maintaining zonation boundaries.

Repopulation of the host liver by transplanted hepatocytes likely requires preferential recruitment to sites of severe damage, in addition to localized expansion. This model is substantiated by three lines of evidence: First, *Afp* ^+^ rHeps during early repopulation (Tr_1W‐3 W) exhibited pronounced activation of promigratory pathways (TGFβ, MAPK, PI3K; Figure 6F), indicating their potential for migration. Second, hepatocytes across Tr_1W‐6 W continuously enriched *Anxa2* ^+^ migratory hepatocyte gene signature [45] without compromising epithelial integrity or polarity (Figure 6G), suggesting a controlled migratory process. Third, by Tr_6 W, the widespread lobular distribution of transplanted hepatocytes was spatially correlated with the maintenance and re‐establishment of cell polarity (Figure 6C), providing direct evidence for successful migration and integration.

Further analysis showed that *Afp* ^+^ rHeps displayed a unique molecular profile, with concurrent elevation of both epithelial and mesenchymal markers and activation of key EMT regulators (Figure 6H). IHC staining confirmed the co‐expression of the epithelial marker CK18 and the mesenchymal marker FN1 in these cells (Figure 6I), demonstrating classic features of epithelial‐mesenchymal plasticity (EMP) [54]. Transcriptomically, this was substantiated by GSEA, which showed these cells were significantly enriched for an established EMP gene signature [54] (Figure 6J). Notably, EMP pathway activity was highest in *Afp* ^low^ cells and decreased progressively in *Afp* ^high^, host, and adult hepatocytes (Figure S8K), establishing the *Afp* ^low^ subset as the principal migratory force.

Collectively, these data establish a division of labor among repopulating hepatocytes: *Afp* ^low^ cells, endowed with heightened EMP activity, serve as the principal migratory engine for parenchymal expansion, while *Afp* ^high^ cells adopt a specialized, Zone3‐like metabolic state for local repair. This coordinated program enables efficient liver repopulation.

## 3 Discussion

Reprogramming of terminally differentiated cells offers a new regenerative paradigm to overcome the limitations faced by cell therapy for liver failure and other end‐stage diseases. Notably, this capacity extends beyond endogenous cells; transplanted cells can also achieve functional conversion within host organ microenvironments [13]. Our prior work demonstrated that mature hepatocytes possess sustained regenerative potential: during 12 rounds of serial transplantations, donor hepatocytes maintained a youthful state, replaced necrotic host hepatocytes, thereby fully restoring liver architecture and function [18]. Here, we show that this capacity arises from adaptive reprogramming into a transient *Afp* ^+^ rHep state, which involves a partial maturation trade‐off to acquire metabolic remodeling capacity and controlled proliferation. This “inverse correlation between proliferation and maturation” mirrors developmental regulation [17, 55], suggesting a conserved biological balance. Critically, this “proliferation‐permissive but differentiation‐restricted” state enables rapid functional recovery after expansion, accounting for the striking repopulation capacity of *Afp* ^+^ rHeps in *Fah* ^−/−^ mice.

In contrast to the bipotent LPLCs generated through endogenous reprogramming in cholestatic injury [9, 10, 28], *Afp* ^+^ rHeps retain unipotent hepatic differentiation potential. This divergence reflects distinct injury contexts: *Fah* ^−/−^ drives hepatocyte‐focused repair, while DDC (the model inducing LPLCs) favors biliary lineage activation. Our cross‐model analysis further reveals that *Afp* ^+^ rHeps represent a functionally optimized endpoint within a shared metabolic landscape of regeneration, with their state defined by coordinated, high‐amplitude activation of the complete energy‐production axis: the PPARγ‐FAO‐TCA‐OXPHOS cascade, uniquely coupled to sustained AFP expression. This metabolically optimized program distinguishes *Afp* ^+^ rHeps from other regenerative populations and underpins their ability to support massive tissue repair, ultimately offering a strategic option that balances safety and efficacy for terminally differentiated cell‐based regenerative therapies.

Having defined this metabolically optimized regenerative state, we identify its central molecular driver. Our data reframe AFP from a passive biomarker [56] into a central effector of liver regeneration. This model is empirically grounded: pharmacologic perturbation confirms that PPARγ activity is both necessary and sufficient for AFP‐driven repair, and a strong positive correlation links *Afp* and *Pparg* expression specifically within the *Afp* ^+^ rHep population. We establish that AFP sustains hepatocyte repair through a positive feedback loop that amplifies PPARγ signaling. This feedforward regulatory axis is indispensable for mediating the pro‐regenerative functions of AFP. Consequently, the amplified PPARγ signaling drives the coordinated PPAR‐FAO‐TCA‐OXPHOS metabolic program, endowing transplanted hepatocytes with the energy and plasticity to adapt to injury.

A critical determinant of AFP function is its regulatory context, not merely its expression. Although both donor‐derived *Afp* ^+^ rHeps and host hepatocytes transcribe *Afp*, their regulatory networks diverge fundamentally. In donor cells, AFP occupies a hub position within a coherent pro‐regenerative module, directly coupling PPARγ signaling to proliferation. In host cells, however, AFP resides peripherally in a network associated with senescence and inflammation, where PPARγ activation is uncoupled from regeneration. This topological disparity, compounded by the host *Fah* ^−/−^ genotype that impairs intrinsic repair, explains why similar *Afp* transcription leads to opposing outcomes, thereby resulting in productive reprogramming in donor cells but regenerative stagnation in host hepatocytes.

Therapeutically, modulating the PPARγ/AFP axis offers dual advantages. Recombinant AFP not only enhances donor hepatocyte repopulation in *Fah* ^−/−^ mice but also improves survival in models of acute liver injury, confirming a generalizable pro‐repair mechanism. Building upon its cell‐autonomous pro‐repair function identified here, AFP can leverage its well‐documented immunosuppressive properties [57, 58, 59]. This synergy allows a single agent to address the twin barriers of poor cell survival and host immunity. Consequently, our findings provide a mechanistic rationale for targeting the PPARγ/AFP axis to concurrently overcome the dual barriers of cell survival and immune rejection, outlining a translatable strategy for improving cell‐based liver repair.

Building on these therapeutic implications, a critical consideration for successful clinical translation lies in the potential impact of standard immunosuppressive regimens on this newly identified regenerative axis. In clinical hepatocyte transplantation, immunosuppression is essential to prevent allograft rejection. However, drugs such as corticosteroids or calcineurin inhibitors may inadvertently suppress neutrophil function or TNFα production [60]. This raises a critical unresolved question: could such regimens dampen the neutrophil‐mediated mitogenic signal crucial for donor hepatocyte repopulation? Alternatively, the PPARγ/AFP and TNF‐α/AP‐1 pathways may represent a distinct and preservable therapeutic axis. Future clinical strategies could, therefore, aim to design immunomodulatory protocols that selectively mitigate rejection while sparing or even augmenting this specific pro‐regenerative immune niche. Resolving this interplay between iatrogenic immunosuppression and physiologically driven regeneration will be vital for optimizing the efficacy of cell‐based therapies and advancing their clinical application in patients.

Beyond molecular and immunological regulation, spatial coordination also plays a critical role in the regenerative process of transplanted hepatocytes. Spatially, our findings indicate that TGF‐β signaling orchestrates the migration of transplanted hepatocytes through EMP induction, enabling them to navigate sinusoids and achieve parenchymal localization. Critically, PPAR‐mediated metabolic remodeling coordinates both global reprogramming and the spatial regulation of zonation‐specific enzymes, guiding localized hepatocytes to acquire molecular signatures aligned with native lobule architecture. These processes suggest a sequential “metabolic‐spatial” coordination program, where physical localization via EMP precedes microenvironment‐adapted functional maturation through PPAR‐mediated signaling. This temporal logic may explain why transcriptional recovery lags behind histological repair after injury [61].

Our analysis further reveals a functional division within the *Afp* ^+^ rHep population. Cells with lower AFP expression display enhanced EMP pathway activity and an intermediate zonation identity, while *Afp* ^high^ cells acquire a transcriptional profile that converges with that of central vein hepatocytes. Although these observations warrant further experimental confirmation, they point toward a compelling biological model: the metabolic divergence characterized earlier may prefigure a functional specialization during repopulation, with one subset potentially dedicated to migration and parenchymal expansion, and the other to local metabolic detoxification.

Our results demonstrate that the proliferative activation of transplanted hepatocytes is tightly coupled to dynamic host immune responses. Infiltrating neutrophils emerge as key niche cells, directly triggering *Afp* ^+^ rHep proliferation through TNF‐α secretion. This finding extends the established paradigm of neutrophil‐derived HGF in promoting hepatocyte proliferation [62], collectively underscoring their non‐redundant role in regeneration. Notably, the pro‐proliferative function of TNF‐α corroborates its documented capacity to sustain long‐term hepatocyte expansion in 3D culture [7], solidifying its status as a central mitogenic regulator. Moreover, neutrophils co‐deliver OSM signals, which help preserve hepatocyte identity by suppressing of biliary transcription factors such as SOX9 and KLF5 [48]. The synergistic interplay between TNF‐α and OSM thus establishes a pro‐regenerative niche that simultaneously drives proliferation and safeguards differentiation. This mechanism highlights the post‐transplant immune microenvironment as a novel therapeutic target, offering a strategy to achieve coordinated expansion and functional preservation of transplanted hepatocytes.

We further identify the AP‐1 transcription factor family as pivotal downstream effectors of TNF‐α signaling that drive proliferative activation in *Afp* ^+^ rHeps. The sequential engagement of this AP‐1‐dependent regeneration response program [46], coupled with their metabolically optimized state, demonstrates that these cells retain an intrinsic capacity for complete parenchymal restoration, effectively bypassing fibrotic scarring. Critically, the *Fah* ^−/−^ model served as the essential discovery platform that enabled the identification and mechanistic dissection of this functionally optimized regenerative module. Its distinctive microenvironment performs a dual role: it enriches hepatocyte subsets with high intrinsic regenerative potential (phenotypically manifested as *Afp* ^+^ rHeps) while concurrently providing the physiological context required to delineate the full spectrum of molecular pathways, from metabolic reprogramming to immunemediated proliferation, that underpin massive repopulation. Collectively, our findings provide deeper mechanistic insights into liver regeneration and, more importantly, establish a novel paradigm for selecting therapeutic hepatocytes based on an integrated signature of metabolic fitness and proliferative competence, rather than mere marker expression.

### 3.1 Limitations of Study

A previous study on a periportal injury‐induced liver regeneration model established that hepatocytes can undergo reprogramming within one week after liver injury [10]. Our results showed that nearly all tdTomato^+^ donor hepatocytes convert into *Afp* ^+^ rHeps within 1 W post‐transplantation, highlighting the critical role of early regulatory events in driving the donor cell phenotypic transition. However, several technical and methodological constraints should be acknowledged. First, the extremely limited number of transplanted hepatocytes in the host liver during this early phase has posed challenges for tdTomato‐based single‐cell analysis. Second, although samples were collected at 3 W post‐transplantation, scATAC‐seq did not robustly detect *Afp* ^high^ donor‐derived cells, constraining our mechanistic understanding of AFP regulation. Third, a key limitation is the correlative nature of the evidence linking *Afp* ^+^ rHeps to repopulated hepatocytes. Our study lacks definitive lineage‐tracing data from an AFP‐Cre model, which would be required to formally establish the direct precursor‐product relationship. These technical constraints may be addressed in future studies with more advanced single‐cell sequencing technologies, optimized protocols, and dedicated AFP‐Cre lineage‐tracing experiments.

The mechanistic insights of this study are primarily derived from mouse models. While core liver regeneration principles are conserved between mice and humans, species‐specific differences may exist in AFP expression patterns, immune microenvironment composition, and metabolic pathway details. Thus, caution is warranted when extrapolating our conclusions to human clinical practice, and validation in human hepatocytes or organoid models will be critical for clinical translation. It is also important to note that AFP functions as a “dual‐faced” molecule: it is a key regulator of physiological regeneration but is also highly expressed in hepatocellular carcinoma. The proliferation‐promoting and metabolic remodeling capacities of AFP, which are beneficial for regeneration, could pose tumorigenic risks if its activation becomes sustained or spatiotemporally dysregulated. Future development of AFP‐targeted regenerative therapies must prioritize precise spatiotemporal control of AFP activity and rigorous long‐term safety assessments.

## 4 Conclusion

This study establishes a mechanistic framework for how transplanted mature hepatocytes regenerate the liver through reprogramming into a transitional *Afp* ^+^ rHep state, providing direct functional evidence for a novel regenerative therapy based on terminally differentiated cells. We demonstrate that AFP, as a core regulator, drives metabolic adaptation via the PPARγ signaling pathway, enabling transplanted cells to thrive in the injured microenvironment, a mechanism validated by both agonist/antagonist studies and its broad hepatoprotective effects across multiple injury models. Furthermore, we delineate a coordinated pro‐regenerative niche: neutrophil‐derived TNF‐α activates the AP‐1 regulon to specifically promote the proliferation of receptor‐high *Afp* ^low^ rHeps, while OSM signaling helps maintain hepatocyte identity. The functional necessity of both the PPARγ/AFP axis and the TNF‐α/AP‐1 pathway is confirmed by direct perturbation experiments in vivo. Collectively, these findings advance a functionally‐validated model for how mature hepatocytes drive liver regeneration, providing a framework and identifying key targets for future therapeutic development.

## 5 Materials and Methods

### 5.1 Mice

All animal experiments were conducted at Tongji University and were approved by the Ethics Committee of Shanghai East Hospital, Tongji University (Approval Number: 2021DYYSD141). Mice were housed under specific pathogen‐free (SPF), temperature‐ and light‐controlled conditions in the animal facility of Tongji University, which is accredited by the Shanghai Science and Technology Committee. Briefly, *Fah* KO mouse was backcrossed in the background of C57 BL/6, and was maintained on 7.5% NTBC. Rosa26‐LSL‐tdTomato was purchased from Jackson laboratory. All mouse in this study were provided free access to food and water and maintained in a standard pathogen‐free environment with a 12/12 h light/dark cycle.

### 5.2 AAV Injection

The Adeno‐Associated Virus (AAV) used in this study was purchased from Genomeditech company (Shanghai, China). Briefly, AAV were produced with plasmids containing the full TBG promoter, which is specifically active in hepatocytes. Through tail vein injection at 5 × 10^10^ copies of the AAV8‐TBG‐Cre virus per mouse. TBG promoter will control the expression of Cre, which specific labeling mature hepatocytes with Tdtomato fluorescence.

### 5.3 Hepatocytes Isolation for Transplantation, Cell Culture, scRNA‐seq, and scATAC‐seq

The primary hepatocytes were isolated by the two‐step collagenase perfusion method established previously [63, 64, 65]. Briefly, mice were anesthetized with tribromohexanol intraperitoneal (150 µL/10 g weight). The abdomen was then exposed, and the inferior vena cava was cut and the liver was perfused with preheat 37°C liver perfusion buffer (buffer I) for 5–10 min followed by liver perfusion II (EBSS+10 mm HEPES+6000 units of collagenase IV) for about 20 min, and then, the liver was transferred into clean dish with DMEM medium containing 10% FBS at 4°C. The liver was separated gently to help the hepatocytes dissociation, and the cell suspension was passed through a 70 and 40 mm cell strainer (BD Falcon), followed by centrifugation at 50 g for 2 min at 4°C. Cell suspension was collected for FACS analysis, single‐cell RNA sequencing, or single‐cell ATAC sequencing.

### 5.4 Spleen Injection and Serial Transplantation

Method was performed as previously described [21, 23, 64, 66]. Briefly, purified hepatocytes were injected into the spleens of *Fah* KO mice, and NTBC was withdrawal two days before cell transplantation. Body weight was recorded every 3 days. During serial transplantation, we performed 2 rounds of cell transplantation, and in the first round, the cell source came from the primary hepatocytes of Rosa26‐LSL‐tdTomato mice with AAV injection. Cell source of the second round came from the round 1 mouse after 12 weeks repopulation. To investigate the impact of AP‐1 signaling on the effectiveness of hepatocyte transplantation, AP‐1 signaling inhibitor (T‐5224, 30 mg/kg) was intraperitoneally injected twice a week after hepatocyte transplantation.

### 5.5 Acute Liver Failure (ALF) Model

After 14 h of starvation, 8‐week‐old male C57BL/6 mice administered intraperitoneal APAP or CCl4 solutions at appropriate concentrations to induce acute liver injury. Tissue samples were collected at designated timepoints for subsequent testing. Mice in the rAFP‐treated group received a single tail vein injection of the corresponding concentration of rAFP before intraperitoneal administration of either APAP or CCl_4_ solution.

### 5.6 Hepatocyte Cultures

After flow cytometry sorting, the host liver cells undergo a brief 2D culture in vitro to observe their proliferation status and the expression efficiency of tdTomato in vitro. Specific liver cell culture methods have been established in previous studies [21, 23]. The cells with a concentration of 2 × 10^5^/mL were taken out and mixed with Matrigel gel (dilution ratio 1:50), and the liquid volume of each well was about 2 mL. The well plates were placed in a 5% CO_2_ incubator for 1 to 2 h, and the cells were attached before the subsequent operation. In order to explore the association between PPARγ and AFP, cells were exposed to rosiglitazone (10 µm), GW9662 (10 µm), and rAFP (200 ng/mL) according to the experimental group after adhesion. Cells were collected after 24 h to allow for RNA extraction for subsequent detection.

### 5.7 Tissue Embedding and Immunohistochemistry Staining

Tissues, including the livers and kidneys of host mice were collected and fixed in 10% formalin for 72 h at room temperature and embedded in paraffin until the sectioning step. Detailed protocol of immunohistochemistry staining was described in previous publications [21, 23, 63, 65, 67, 68, 69]. For immunostaining, tissue sections were blocked with PBST (0.2% Triton X‐100+10% normal donkey serum+1% BSA) for 40 min at room temperature, followed by primary antibody incubation overnight at 4°C. The next day, tissue sections were washed with PBS three times and incubated with fluorescence conjugated secondary antibodies (Abcam) for 40 min at 37°C, then washed with PBS three times and mounted with antifade mounting medium.

Primary antibodies used in this study included: tdTomato (Arigo, ARG55724, 1:400 dilution), AFP (Proteintech, 14550‐1‐AP, 1:200 dilution), E‐CAD (BD biosciences, 610181, 1:50 dilution), GS (Abcam, ab49873, 1:1000 dilution), Ki67(Abcam, 16667, 1:200 dilution), HNF4a (Abcam, 201460, 1:200 dilution), FAH (Cell Lab Tech, CLT‐602‐910, 1:3000 dilution), Images were acquired using a Leica microscope (m205), and were analyzed by the Image J.

### 5.8 Rt‐Pcr

RNA was extracted by TRizol method. The reverse transcription system was conducted as follows: 2 µL of 5×RNA reverse transcriptase, 300–500 ng of RNA, and DEPC water were supplemented to a final volume of 10 µL. The reaction conditions included incubation at 37°C for 15 min followed by heating at 85°C for 15 s. The resulting cDNA obtained through reverse transcription was stored long‐term at ‐80°C.

### 5.9 Hematoxylin & Eosin Staining

The mice were euthanized, and intact heart tissues were extracted. Tissues were immersed in 10% formalin and placed on a shaker for 24 h to undergo fixation. After fixation, the tissues were dehydrated, embedded, and finally prepared into sections, which underwent heating, deparaffinization, and rehydration processes, followed by three washes with PBST (phosphate‐buffered saline with Tween‐20 detergent), with each wash lasting for 5 min. After washing, the sections were stained with hematoxylin, immersing them in a hematoxylin staining solution typically for 5 min. Then, the sections counterstained by rinsing them repeatedly in running tap water for 5 to 10 min. Afterward, the samples were dehydrated with sequential immersion in 50%, 70%, 80%, and 95% concentrations of ethanol, with each concentration lasting for 2 min, respectively. The sections were stained with eosin by gently applying eosin staining solution for 1 min. After completing the H&E staining process, the obtained results were documented by photography.

### 5.10 Western Blot

Total protein of liver tissues from *Fah* ^−/−^ mice was extracted using RIPA lysis buffer added with protease inhibitor cocktail tablets. Phosphatase inhibitor cocktail and protease inhibitor cocktail were prepared from ApexBio (cat# K1015; K1007), and the doses of the protease and phosphatase inhibitors are diluted at a 1:100 (v/v) ratio. More milk and BSA were used to maximize the reduction of non‐specific binding, thereby improving the signal‐to‐noise ratio and enhancing the specificity and sensitivity of the Western blot assay. Then, the BCA protein quantification kit (P0011, Beyotime, Shanghai, China) was used to measure protein concentration. Next, a 12.5% polyacrylamide gel was produced to separate each protein, and the separated proteins were transferred into PVDF membranes (Millipore, IPVH00010). The membrane was blocked in PBS‐T containing 5% milk/BSA for 2 h before overnight incubation with a primary antibody at 4°C. After 2 h incubation with a secondary antibody, signals were quantitated using an Odyssey infrared imaging system.

### 5.11 Liver Panel Analysis

Blood was collected and stored at 4°C for 1 h. Freshly isolated serum was obtained by centrifuging the blood at 12,000 g for 10 min and stored at ‐80°C before use. ALB, ALT, AST, T‐BIL were detected according to the manufacturer's instructions (Shanghai Shensuo UNF Medical Diagnostic Articles Co., Ltd.).

### 5.12 Immunoprecipitation and Mass Spectrometry

Cell lysates from 5 × 10^6^ cells were prepared using the IP buffer (50 mm Tris pH 7.5, 150 mm NaCl, 1% TritonX‐100, 0.5% Na‐DOC, 1 mm EDTA, 2 mm PMSF, and 1 × Roche protease inhibitor cocktail). AFP antibody (2 µg) was added to IP samples. Reaction was incubated for 1 h at 4°C on a rotator. 25 µL Protein G agarose beads (Roche) were placed into each IP sample and incubated O/N at 4°C on a rotator. Proteins were eluted using 2 × Laemmli buffer (Bio‐Rad) at 95°C for 5 min at 1000 rpm. Proteomics were performed at the Mass Spectrometry (MS) Core Facility at the Beyotime (Shanghai, China).

### 5.13 Single‐Cell RNA Sequencing

Single‐cell suspensions (1 × 10^5^ cells/mL) with PBS (HyClone, USA) were loaded onto microfluidic devices and scRNA‐seq libraries were constructed according to the protocol from GEXSCOPE Single Cell RNA Library Kit (Singleron). Individual libraries were diluted to 4 nm and pooled for sequencing on an Illumina HiSeq X device with 150 bp paired‐end reads.

### 5.14 Single‐Cell ATAC Sequencing

scATAC‐seq library was constructed according to the 10X Chromium Single Cell ATAC Reagent Kits User Guide. Library was paired‐end sequenced on an Illumina NovaSeq 6000 device.

### 5.15 Data Preprocessing of scRNA‐seq and scATAC‐seq

scRNA‐seq reads were processed with celescope (v1.12.0) for quality filtering, barcode demultiplexing, and genome alignment (mm10), generating gene‐by‐cell count matrices. Cells were retained based on: (1) 500–3500 detected genes, (2) mitochondrial fractions < 20% (hepatocytes) or < 5% (NPCs), and (3) expression of tdTomato^−^ *Fah* ^−^ (host‐derived) or tdTomato^+^ *Fah* ^+^ (transplanted) markers. Ambient RNA was removed using decontX (v1.4.1) [70] (contamination score < 0.2). Doublets were filtered using DoubletFinder (v2.0.3) [71] at an expected rate of 0.8% per 1000 cells (nCells × 8 × 10^−6^). The distribution of gene counts, UMI counts, and % mitochondrial content after applying QC cutoffs is presented in Figure S2A.

For scATAC‐seq data, Cell Ranger ATAC (v2.1.0) was used for barcode demultiplexing, genome alignment (mm10), and quality filtering, producing fragment files and cell‐by‐peak matrices. Quality control was performed with Signac (v1.10.0) [72] through calculation of nucleosome signal strength (NucleosomeSignal function) and TSS enrichment scores (TSSEnrichment function), followed by removal of cells with outliers according to established thresholds.

### 5.16 Analysis of scRNA‐seq Data

#### 5.16.1 scRNA‐seq Sample Integration, Clustering, and Annotation

To integrate all scRNA‐seq samples and mitigate batch effects, we performed an integrated analysis using Seurat (v4.3.0) [73]. Following quality control (as described above), cells were normalized and scaled using the NormalizeData, FindVariableFeatures, and ScaleData functions, respectively. For dimensionality reduction, non‐negative matrix factorization (NMF; v0.30.4) [74] was employed instead of conventional PCA, using the top 6000 variable genes identified by the FindVariableFeatures function to better characterize cellular states. Batch effects across samples were then removed using Harmony (v0.1.1) [75] on the NMF‐derived low‐dimensional embeddings. This was followed by cell clustering with the FindClusters function (resolution optimized for each dataset). Final cluster identities were assigned based on canonical marker gene expression patterns.

#### 5.16.2 Differential Expression and Functional Enrichment Analysis

DEGs between pairwise comparison groups were identified using the FindMarkers function (Seurat, v4.3.0) with Wilcoxon rank‐sum tests. Specific significance thresholds were applied for each comparison (detailed in Results). Functional enrichment analysis was performed using clusterProfiler (v4.8.3) [76], with GO analysis conducted via the enrichGO function and pathway analysis implemented through the enrichKEGG function. This identical enrichment pipeline was systematically applied to all subsequent gene set analyses.

#### 5.16.3 Gene Set Activity Scoring and Enrichment Analysis

To evaluate pathway activity and functional signatures, three gene set scoring approaches were utilized:

1. GSEA analysis: Differentially expressed genes between each cell group and the control group (R0_0 W, also denoted as Adult) were identified using the wilcoxauc function in presto (v1.0.0). Genes were ranked in descending order by their AUC values, which were used as the statistics for preranked GSEA using the GSEA function in clusterProfiler (v4.8.3).
2. GSVA analysis: Enrichment scores at the single‐cell level for each sample were calculated using GSVA (v2.0.0), with input data as the normalized expression matrix generated by Seurat (v4.3.0). Parameters were set as method = ‘gsva’ and kcdf = ‘Gaussian’.
3. Module score calculation: Single‐cell level gene signature scores were calculated using the AddModuleScore function in Seurat (v4.3.0) with default parameters.

These approaches were applied to both published and custom‐defined gene signatures or databases. Scores from all methods were normalized across samples or cell groups for comparative analysis.

#### 5.16.4 Pseudotime Analysis

1. For integrated data combining transplanted hepatocytes and developmental hepatocytes (GSE90047 [14], GSE209749 [17]): Sample‐level mean expression values of maturation‐related genes [17] were computed using the AverageExpression function in Seurat (v4.3.0). PCA was subsequently performed on these mean expression values using FactoMineR (v2.8).
2. For integrated data combining transplanted hepatocytes, postnatal hepatocytes, and DDC‐induced reprogrammed hepatocytes (GSE151309 [12], GSE171993 [35], GSE212692 [10]): The top 2,000 HVGs were identified using the FindVariableGenes function in Seurat (v4.3.0). PCA was performed with FactoMineR (v2.8) using normalized expression values of these HVGs. Diffusion maps were generated using the DiffusionMap function in destiny (v3.20.0) [77]. Additionally, major trajectories derived from PCA results and diffusion maps were fitted to a principal curve (with smoothing spline fitness) using the principal_curve function in princurve (v2.1.6), where the arc length along the curve was defined as pseudotime.

#### 5.16.5 Identification of ARS

To characterize the reprogramming process of mature hepatocytes into *Afp* ^+^ rHep, two relevant gene sets were identified: (1) Genes significantly upregulated in *Afp* ^+^ rHep (group.1) compared to group.2 hepatocytes (log_2_FC > 1, adjusted *p* < 0.05); (2) Given that reprogramming is initiated early post‐transplantation, a set of genes upregulated in transplanted 1 and 3 W samples was identified using TCseq (v1.18.0) (Figure S4E). The union of these two gene sets was then defined as ARS. For the complete list of ARS genes, please refer to Table S7. Notably, ARS and RRG [10] share only 12 overlapping genes (Figure S4F).

#### 5.16.6 Metabolic Pathway Analysis

To evaluate metabolic activities at single‐cell resolution, 82 metabolic pathways from the KEGG database were analyzed using GSEA and GSVA approaches. Cell‐specific metabolic fluxes were further estimated using scFEA [36], a graph neural network model that incorporates 22 super module classes containing 169 metabolic modules to quantify metabolic dynamics.

#### 5.16.7 Trajectory Analysis

Developmental trajectories of transplanted hepatocytes were reconstructed using four computational methods: (1) Monocle 2 (v2.30.1) [38] with top 2,000 HVGs selected by the FindVariableFeatures function (Seurat, v4.3.0) and trajectories constructed via DDRTree algorithm; (2) Monocle 3 (v1.0.0) [37] implementing standard pseudotime calculation; (3) scVelo (v0.3.3) [39] for RNA velocity inference in dynamical mode; and (4) Slingshot analysis performed using the RunSlingshot function from SCP (v0.5.6) to reconstruct lineage paths.

#### 5.16.8 Inference of Cell Cycle Phases

To classify hepatocyte cell cycle states, two computational approaches were implemented according to their standard pipelines: (1) ccSeurat, which scores cells based on canonical phase‐specific marker gene expression using the CellCycleScoring function (Seurat, v4.3.0) to assign cell cycle phases; and (2) ccAFv2 [78], leveraging an artificial neural network (ANN) with dropout‐regularized hidden layers to compute state‐specific likelihoods, assigning the most probable phase when exceeding predetermined confidence thresholds.

#### 5.16.9 Gene Co‐Expression Network Analysis

Co‐expression network analysis was performed using hdWGCNA (v0.3.1) [79] to identify gene modules containing *Afp*. For selected *Afp*‐associated modules, *Afp*‐centric sub‐networks were constructed through sequential filtering: genes within these modules that had module eigengene‐based connectivity (kME) > 0.25 were first selected; then, among these, genes directly connected to *Afp* in the topological overlap matrix (TOM) with TOM weight > 0.025 were retained to establish robust functional connections.

#### 5.16.10 Co‐IP‐MS Data Analysis for AFP Interacting Proteins

High‐confidence AFP‐interacting proteins were identified via stepwise filtering of Co‐IP‐MS data: (1) low‐quality proteins (< 2 unique peptides, score < 30, or sequence coverage < 5%) were excluded; (2) non‐specific binders were removed by retaining proteins with FC > 4 (vs. IgG controls) and experimental intensity exceeding mean IgG intensity + 3×SD; (3) known contaminants (e.g., serum/housekeeping/ribosomal proteins) and proteins with iBAQ (%) < 0.1 were further filtered out.

Zero values in iBAQ_IgG were replaced with half the minimum non‐zero value to enable log‐transformation, and log_2_(iBAQ/iBAQ_IgG) was calculated for each protein. Conserved interactors were determined by intersecting results from two independent experiments (Ctl and AFP‐OE), and average log_2_FC and log_2_(iBAQ ratio) values for overlapping proteins were computed as final high‐confidence interactions.

#### 5.16.11 Gene Regulatory Network (GRN) Analysis

GRNs were inferred using pySCENIC (v0.12.1) [80], with DEGs retained and co‐expression modules determined via GRNboost2 (arboreto, v0.1.6). This generated an AUC matrix representing the activity of regulons (TF‐target gene regulatory units). As a complementary approach, regulon activity was also computed using decoupleR (v2.12.0) [81]. To evaluate cell group specificity, a Relative Specificity Score (RSS) was calculated for both datasets. For each regulon, two metrics were first computed: (1) the mean activity in the target cell group (target_activity), and (2) the mean activity in other groups (other_activity). The RSS was derived by normalizing their difference (target_activity—other_activity) against the regulon's maximum activity. This metric, serving as the unified screening criterion, quantifies regulon‐cell group associations, with higher values indicating greater specificity. Through this screening process, TFs regulating *Afp* or ARS genes were identified.

#### 5.16.12 Single‐Cell Ligand‐Receptor Interaction Analysis

Cell‐cell communication networks were inferred using CellChat (v2.2.0) [82], based on ligand‐receptor co‐expression patterns in scRNA‐seq data. The normalized expression matrix and cell type annotations derived from the Seurat object were processed through CellChat's probabilistic framework. Interaction probabilities between cell clusters were computed using the computeCommunProb function with default parameters. These interactions were then mapped to signaling pathways via the computeCommunProbPathway function, utilizing CellChat's built‐in database (CellChatDB v2) with default settings. While this approach identifies statistically significant interactions, it is inherently limited by transcriptional noise and cannot account for spatial constraints or low‐abundance paracrine signals.

#### 5.16.13 Construction of a Classifier for Hepatocyte Zonation Prediction

To predict lobule layers of hepatocytes, an XGBoost classification model was developed through the following steps: (1) Training dataset: processed genes × cells matrix derived from scRNA‐seq profiles (GSE84498 [41]), where cells were pre‐annotated into 8 layers (labels 1–8) representing zonation classes (Figure S8B); (2) Feature selection: from 18 well‐known zonation‐related genes [41], the top 4 genes (*Cyp2f2*, *Cyp2e1*, *Glul*, and *Ass1*) were selected as features based on mean absolute SHAP values (SHAP, v0.44.1) (Figure S8E). The corresponding sub‐matrix was extracted; (3) Model training: the classification model was built using XGBoost (v1.6.2) [83] with hyperparameters optimized via Optuna (v4.4.0) [84] through 500 trials (TPE sampler and median pruner). Workflow implementation relied on scikit‐learn (v1.3.2), including sample weight calculation (compute_class_weight function, to address class imbalance), stratified 5‐fold cross‐validation (StratifiedKFold function, for performance evaluation), and pipeline integration (Pipeline function). This approach yielded a mean multiclass AUC of 0.974 (Figure S8F,G). Additionally, the trained model was applied to predict layers (L1‐L8) for hepatocytes in the R0_0 W sample (used as an independent test set), as the layer annotations generated using the method of Droin et al. [85]. failed to meet expectations, thereby motivating the development of our new predictive framework (Figure S8C,D,H). For comparison with liver zonation staining results, L1‐L3 were grouped as Zone3, L4‐L6 as Zone2, and L7‐L8 as Zone1.

#### 5.16.14 Data Imputation for Visualization

To better visualize gene expression on scRNA‐seq maps, data imputation was performed using Rmagic (v2.0.3) [86] with default parameters. The underlying data analysis was conducted on unimputed data, and this imputation was used solely in scRNA‐seq visualizations.

### 5.17 Analysis of scATAC‐seq Data

#### 5.17.1 Integration of scATAC‐seq Datasets

To integrate two scATAC‐seq datasets (Tr_3 W and Adult (GSE158873 [87])), we performed the following steps using Signac (v1.10.0): (1) A common peak set between the two datasets was first identified; (2) Peak counts for this common set in each sample were then re‐quantified using the FeatureMatrix function; (3) The two datasets were merged, followed by normalization using the RunTFIDF function, selection of variable peaks with the FindTopFeatures function, and dimensionality reduction via the RunSVD function to generate latent semantic indexing (LSI) dimensions; (4) Batch effects across samples were corrected using the RunHarmony function (Harmony, v0.1.1) on the LSI dimensions; (5) The RunUMAP function (Seurat, v4.3.0) was applied to the harmonized LSI dimensions to compute the final UMAP embedding.

#### 5.17.2 Cell Type Annotation

Cell type annotation was performed by transferring labels from matched scRNA‐seq data to scATAC‐seq data using Signac (v1.10.0) and Seurat (v4.3.0): (1) scATAC‐seq peaks were transformed to a gene activity matrix using the GeneActivity function; (2) Cross‐modal anchors between scATAC‐seq and scRNA‐seq datasets were identified using the FindTransferAnchors function, with scRNA‐seq data as the reference; (3) Cell type labels for scATAC‐seq cells were predicted using the TransferData function.

#### 5.17.3 Differential Accessibility and Transcription Factor Motif Analysis

All analyses were performed using Signac (v1.10.0) as follows:

1. Differential accessible peaks (DAPs) between Tr_3 W and Adult hepatocytes were identified using the FindMarkers function. DAPs were defined as peaks meeting stringent thresholds of absolute log_2_ fold change > 1 and FDR < 0.001 (Benjamini‐Hochberg correction).
2. TF binding motifs were identified in accessible peaks using the AddMotifs function. The JASPAR2022 CORE collection (vertebrate subset, non‐redundant versions only) was used as the motif database, with the mm10 mouse genome assembly as the reference. GC content correction was applied to control for sequencing bias.
3. Motif enrichment analysis for DAPs was performed using the FindMotifs function, which identifies overrepresented TF binding motifs within the differential peaks relative to the default background set of all accessible peaks. Motifs with FDR <0.05 (Benjamini‐Hochberg correction) were considered significantly enriched.
4. Footprinting analyses were conducted to assess direct TF‐DNA interactions using the Footprint function (500‐bp windows centered on motifs, Tn5‐corrected), with the mm10 mouse genome assembly as the reference. Specifically, this function identifies regions of reduced accessibility (footprints) within TF binding motifs, which are indicative of direct protein‐DNA interactions.

### 5.18 Analysis of Bulk ATAC‐seq

Raw reads taken from CNP0000198 (homeostatic liver ATAC‐seq) [88] and GSE142089 (E16.5 and 17.5 HC ATAC‐seq) [15] were aligned to the mm10 mouse genome assembly using Bowtie2 (v2.5.4) [89] with the settings ‘–very‐sensitive’. Alignments were converted to BAM format, sorted, and filtered as follows: (1) Low‐quality reads (mapping quality score (MAPQ) < 30) and mitochondrial reads (chrM) were removed using samtools (v1.21) [90]; (2) PCR duplicates were removed using sambamba (v1.0.1) [91]; (3) Reads overlapping ENCODE mm10 blacklist regions were excluded using bedtools (v2.31.1) [92]. Filtered BAM files were sorted and indexed with samtools (v1.21), followed by removal of intermediate files. Read coordinates were adjusted for ATAC‐seq fragment bias using alignmentSieve (deepTools, v3.5.6) [93], and the resulting BAM files were re‐sorted and indexed. Accessible chromatin regions (peaks) were identified by MACS3 (v3.0.3) [94] with a significance threshold of *p* < 0.05. Motif analysis for ATAC‐seq peaks was performed by motifmatchr (v1.28.0).

### 5.19 Statistical Analysis

SPSS 25 and GraphPad 9 were used for statistical analysis and mapping of the data generated in this experiment. The measurement data were described by mean ± standard deviation. Student's t‐test was used to compare samples between two groups, and bidirectional analysis of variance (ANOVA) was used to compare samples between multiple groups. Kaplan‐Meier curve was used for survival analysis, and Log‐Rank test was performed. *p* < 0.05 was considered statistically significant.

All sequencing data generated in the study will be publicly available as of the date of publication. This paper does not report original code. Any additional information required to reanalyze the data reported in this paper is available from the lead contact upon request.

## Author Contributions

Z.Y.H. designed the study. T.F. performed bioinformatic analysis. C.Y., H.Q., and Y.D. performed experiments. T.F., C.Y., W.C.Z., and Z.Y.H. wrote the manuscript with help from the other authors. Z.Y.H. and W.C.Z. supported and supervised the study. All authors read and approved the final manuscript.

## Conflicts of Interest

The authors declare no conflicts of interest.

## Supporting information




**Supporting File 1**: advs73878‐sup‐0001‐SuppMat.docx.

**Supporting File 2**: advs73878‐sup‐0002‐Tables.xlsx.

## Data Availability

The data that support the findings of this study are available from the corresponding author upon reasonable request.
