# Supplementary material for: Conversion of Transplanted Mature Hepatocytes into Afp + Reprogrammed Cells for Liver Regeneration After Injury
Source: Adv Sci (Weinh). 2026 Jan 29;13(18):e17126. doi: 10.1002/advs.202517126 (PMC13042875; doi:10.1002/advs.202517126)
Supplement: Supplementary file 1 — Supporting File 1: advs73878‐sup‐0001‐SuppMat.docx. [file ADVS-13-e17126-s001.docx]

**Supplemental Information**

**Conversion of Transplanted Mature Hepatocytes into *Afp*^+^ Reprogrammed Cells for Liver Regeneration after Injury**

Ting Fang, Chao Yang, Hua Qiu, Yuan Du, Xicheng Wang, Yuting Li, Mingyang Xu, Changcheng Liu, Xiuhua Li, Na Guo, Jun Shi, Wencheng Zhang, Zhiying He

**Supplementary Figures**


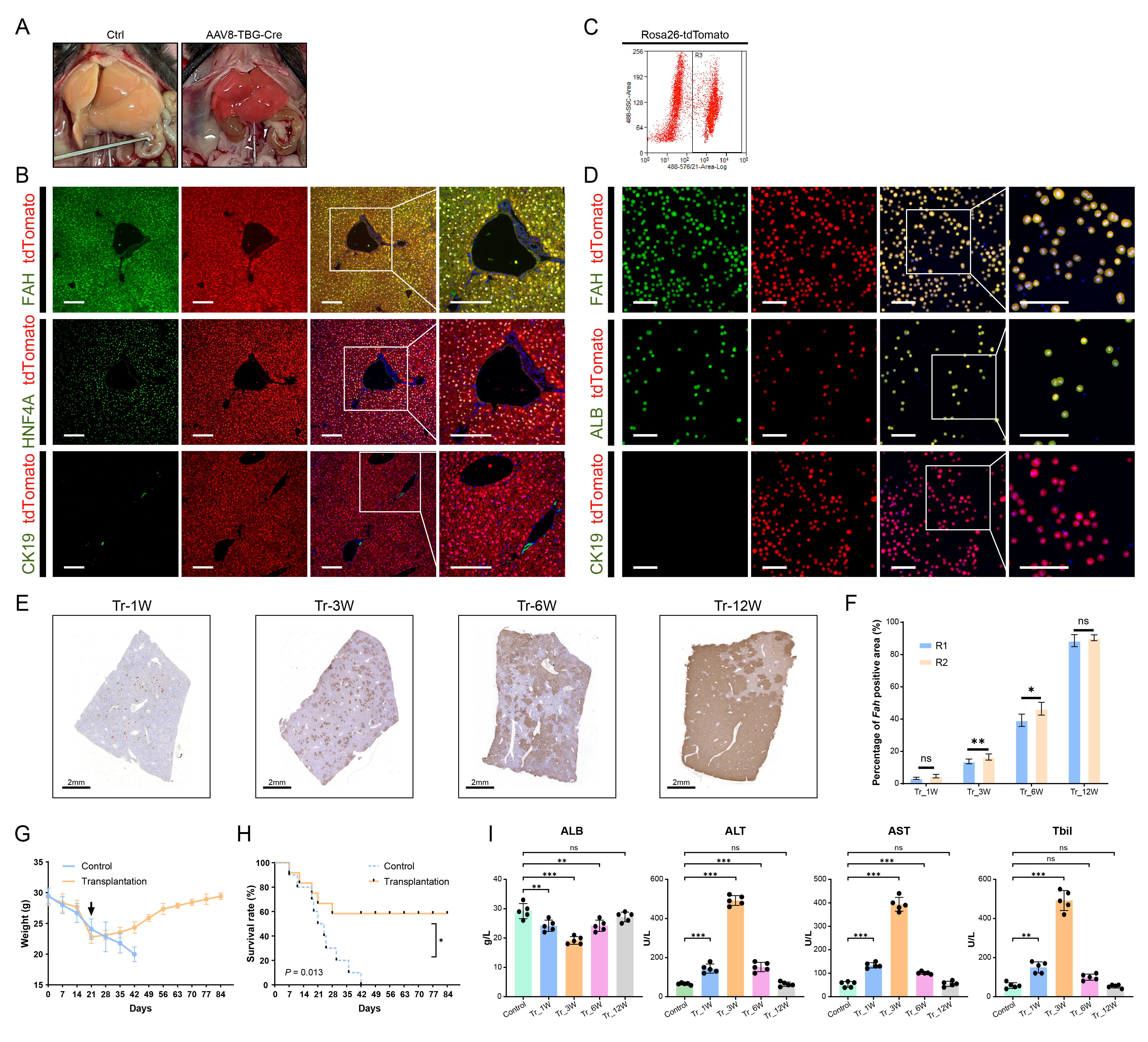


Figure S1. Transplanted mature tdTomato^+^ hepatocytes repopulate damaged host livers in 12 weeks. Related to Figure 1

1. Gross anatomy of a healthy donor Rosa26-LSL-tdTomato mouse liver (**left**). The liver of a Rosa26-LSL-tdTomato mouse injected with TBG adeno-associated virus (AAV8-TBG-Cre) appears uniformly red after 3 weeks of single-dose injection (**right**).
2. Immunofluorescence (IF) co-staining of tdTomato with FAH (**top**)/HNF4A (**middle**)/CK19 (**bottom**) in AAV8-TBG-Cre injected Rosa26-LSL-tdTomato mouse liver sections. Scale bars: 100 μm.
3. Flow cytometry assay hepatocytes isolated from the Rosa26-LSL-tdTomato mice transfected with AAV8-TBG-Cre for 3 weeks. More than 65% of these hepatocytes were tdTomato^+^.
4. IF staining of FAH (**top**), ALB (**middle**) and, CK19 (**bottom**) of purified tdTomato^+^ hepatocytes. Scale bars: 100 μm.
5. Immunohistochemistry (IHC) staining of FAH in *Fah*^-/-^ host livers at 1, 3, 6, and 12 weeks after the transplantation of 1x10^6^ tdTomato^+^ hepatocytes via spleen. Brown cytoplasmic staining indicated FAH^+^ donor cells. Scale bars: 2 mm.
6. The statistical analysis of two rounds of hepatocyte transplantation. The x-axis indicates different timepoints after transplantation, and the y-axis indicates the repopulation index (the ratio that the donor cells have replaced the injured host hepatocytes).
7. The changes of body weight of NTBC-withdrew *Fah*^-/-^ mice in the hepatocyte transplantation group (n=27) and in the control group without treatment (n=20).
8. Survival curve for the mice in the transplantation group (n=20) and in the control group (n=18). **P* < 0.05.
9. Changes of liver serological indexes (ALB, AST, ALT and TBIL) of host mice at different timepoints after tdTomato^+^ mature hepatocyte transplantation (n=5). ns: not significant, **P* < 0.05, ***P* < 0.01, ****P* < 0.001.


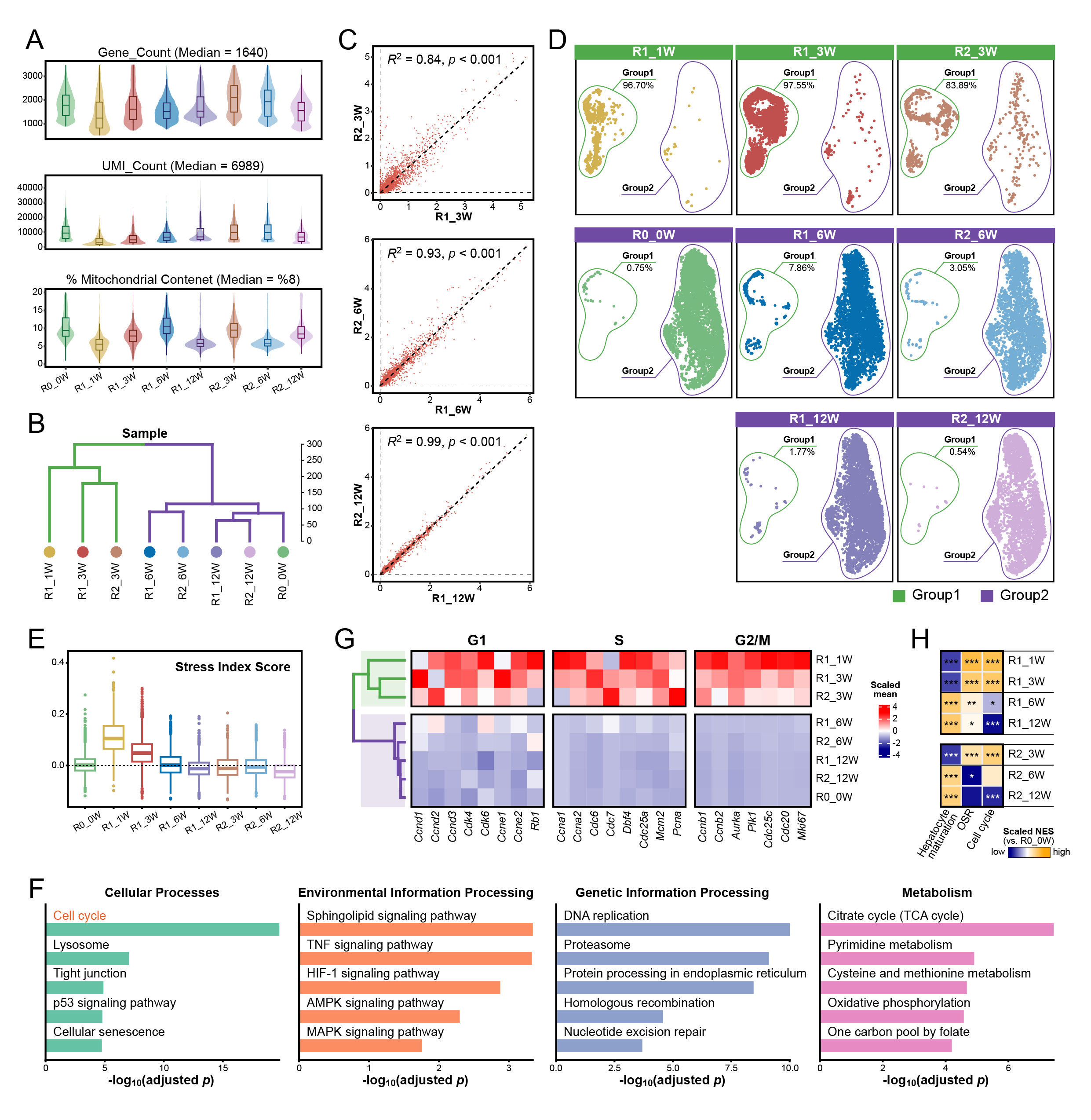


**Figure S2. Quality assessment of scRNA-seq data and characterization of transplanted hepatocytes. Related to Figure 1.** **Additional details are provided in Table S1.**

1. Violin plots of key quality control metrics across eight samples: number of detected genes (median 1,640), unique molecular identifier (UMI) counts (median 6,989), and percentage of mitochondrial reads (median 8%).
2. Hierarchical clustering tree indicating the correlations between samples based on expression levels of differential genes. Colors (green and purple) distinguish two groups of samples.
3. Scatter plots show the correlation between samples from two rounds of hepatocyte transplantation, based on average gene expression.
4. Uniform manifold approximation and projection (UMAP) visualization of hepatocytes from eight samples, colored by sample origin. Green and purple correspond to samples contributing to Group1 and Group2, respectively.
5. Boxplot of the "Stress Index Score"^[^[^1^](#_ENREF_1)^]^ for individual cells, grouped by the eight samples.
6. Bar plots of the top 5 Kyoto Encyclopedia of Genes and Genomes (KEGG) pathways (adjusted *p* < 0.05) within each major category defined in **Figure 1E**. The x-axis represents the -log_10_(adjusted *p*) from the enrichment analysis.
7. Heatmap of cell cycle phase-specific gene (G1, S, G2/M) expression in transplanted hepatocytes at each timepoint.
8. Heatmap showing the NES of hepatocyte maturation, oxidative stress response (OSR), and cell cycle signatures for each transplant timepoint relative to R0_0W. Asterisks denote pathways that are significantly different from R0_0W (*adjusted *p* < 0.05, **adjusted *p* < 0.01, ***adjusted *p* < 0.001).

**
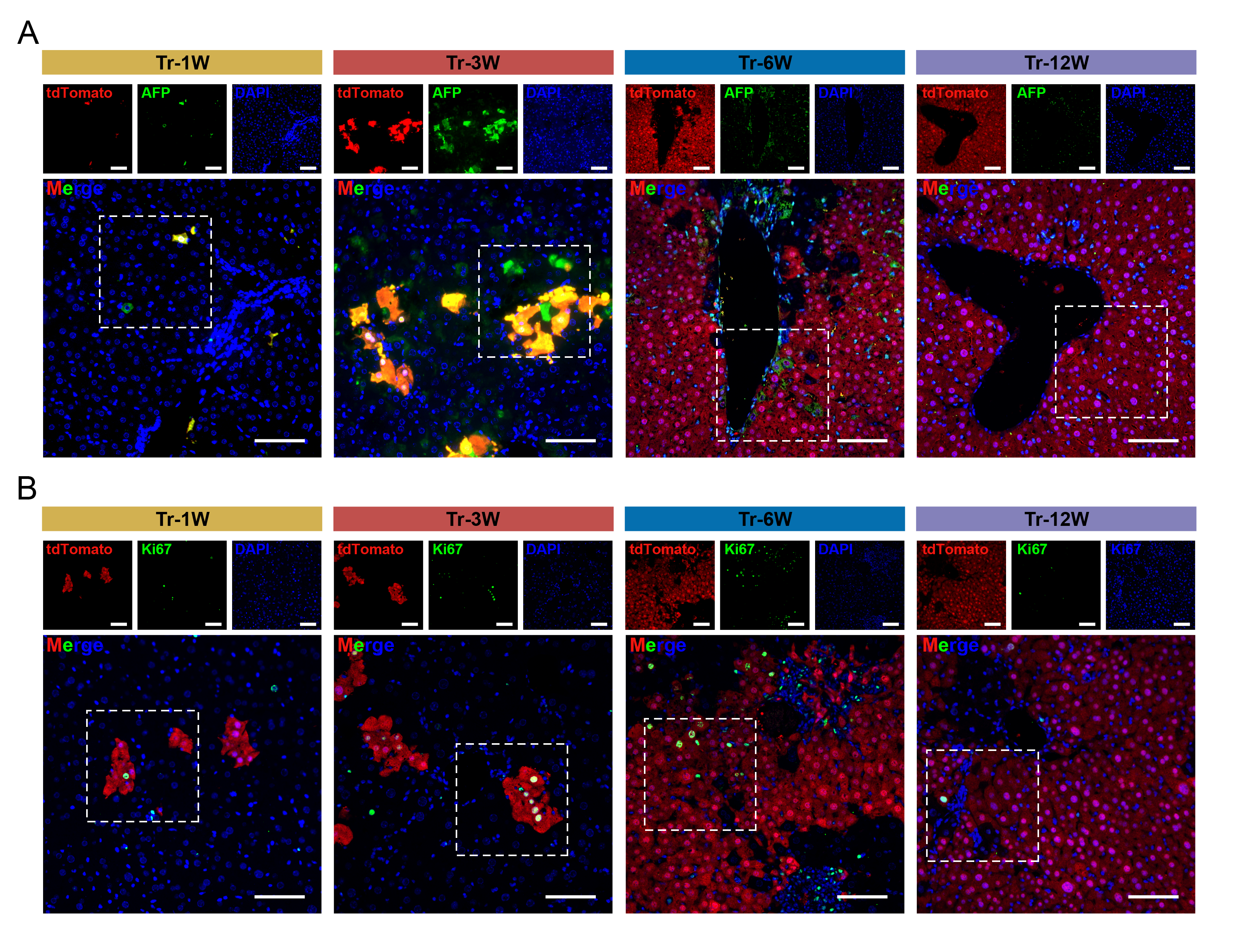
**

**Figure S3. Proportion and distribution of tdTomato^+^AFP^+^ and tdTomato^+^Ki67^+^ hepatocytes in host livers at four timepoints post-transplantation.** **Related to Figure 1**

Multicolor IF staining of host livers at four timepoints post-transplantation (Tr-1W, 3W, 6W and 12W): (**top-left**) Red fluorescence marks the tdTomato^+^ transplanted hepatocytes; (**top-middle**) Green fluorescence indicates (**A**) the AFP^+^ hepatocytes (including host and transplanted hepatocytes) or (**B**) the Ki67^+^ cells (including host cells and transplanted liver cells); (**top-right**) Blue fluorescence (DAPI) labels the cell nuclei; (**bottom**) Merged channels (tdTomato/AFP/Ki67/DAPI) from panels above. **Figure 1G** (**left**) and **Figure 1H** (**left**) are enlarged areas of the selected fields of view in **A** (**bottom**) and **B** (**bottom**), respectively. Scale bar:100 μm.


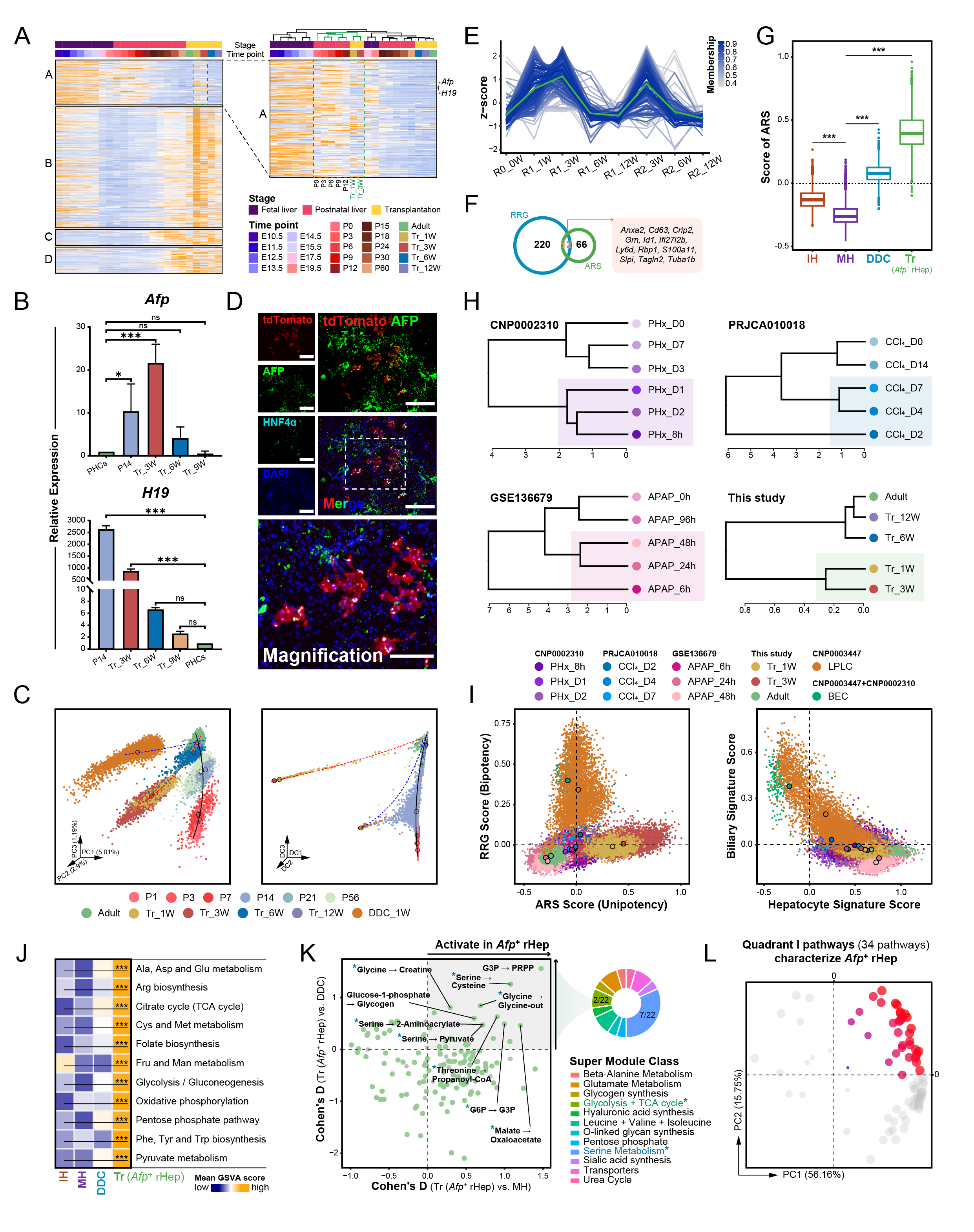


**Figure S4. Analysis of the differentiation status of transplanted hepatocytes. Related to Figure 2.** **Additional details are provided in Table S2.**

1. Heatmap (**left**) of the mean expression of maturation-related genes^[^[^2^](#_ENREF_2)^]^ in hepatocytes from the 23 timepoints in **Figure 2A**. Hierarchical clustering (**right**) of timepoints based on Cluster A gene expression reveals similar expression patterns between Tr_1-3W and P0-P12. Each column represents a timepoint.
2. Quantitative RT-PCR (qRT-PCR) analysis of mRNA levels for two feature genes in three cell types: primary hepatocytes (PHCs), P14 hepatocytes, and hepatocytes at 3, 6, and 9 weeks post-transplantation. ns: not significant, **P* < 0.05, ***P* < 0.01, ****P* < 0.001.
3. 3D-principal component analysis (PCA) plot (**left**) and 3D-diffusion map (**right**) each visualize the developmental progression of hepatocytes across 12 timepoints, including DDC_1W not analyzed in **Figure 2B-C**. In both panels, cells are colored by timepoint. Inferred trajectories for maturation (black), transplantation (red), and DDC response (blue) are overlaid.
4. Multiplex IF staining for tdTomato, AFP and HNF4α in host livers at 3 weeks post-transplantation. Scale bars:100 μm.
5. Line plot showing genes that are upregulated in the early stages of repopulation (R1_1W, R1_3W, and R2_3W). The y-axis indicates the z-score expression. The color gradient represents the magnitude of the gene set membership score for each gene.
6. Venn diagram illustrating the overlap between reprogramming-related genes (RRG)^[^[^3^](#_ENREF_3)^]^ and ARS. The intersection contains only 12 genes.
7. Boxplot of the *Afp*^+^ rHep-related signature (ARS) module scores for individual cells, grouped by the four clusters defined in **Figure 2F**. Asterisks denote significant increases relative to the MH group (two-sided Wilcoxon rank sum test, ***adjusted *p* < 0.001).
8. Selection of regeneration timepoints from four injury or repair models. Dendrograms of hepatocyte samples (PHx, CCl₄, APAP, transplantation) from scRNA-seq data reveal a temporal progression. For each model, timepoints forming branches distinct from their respective baseline controls (PHx_D0, CCl₄_D0, APAP_0h, Adult) were selected to represent active regeneration phases for metabolic analysis.
9. Dual-parameter profiling of hepatocyte states across regeneration models and reference populations. (**Left**) Scatter plot of ARS score (x-axis) versus RRG score (y-axis). (**Right**) Scatter plot of hepatocyte signature score (x-axis) versus biliary signature score (y-axis). Analysis includes hepatocytes from selected regeneration timepoints (identified in **Figure S4H**), LPLCs, and BECs. Points represent individual cells, colored by experimental group.
10. Average GSVA scores across all groups in **Figure 2F** for Tr-specific upregulated metabolic pathways. Asterisks mark pathways that are significantly upregulated in the Tr group relative to each of the other groups individually (two-sided Wilcoxon rank sum test, ***adjusted *p* < 0.001).
11. Scatter plot (**left**) comparing the effect size (Cohen's d) of metabolic flux differences (predicted by scFEA^[^[^4^](#_ENREF_4)^]^) across two comparisons (Tr vs. MH and Tr vs. DDC). Donut chart (**right**) quantifying the proportions of super metabolic module classes enhanced in the Tr group.
12. Pathway-level PCA revealing which pathways drive the sample distribution in **Figure 2L**. Analysis of 82 KEGG pathways highlights those in Quadrant I (high PC1, high PC2) as key to the unique metabolic state of *Afp*^+^ rHeps, with point size and color indicating each pathway's overall influence (distance from origin).


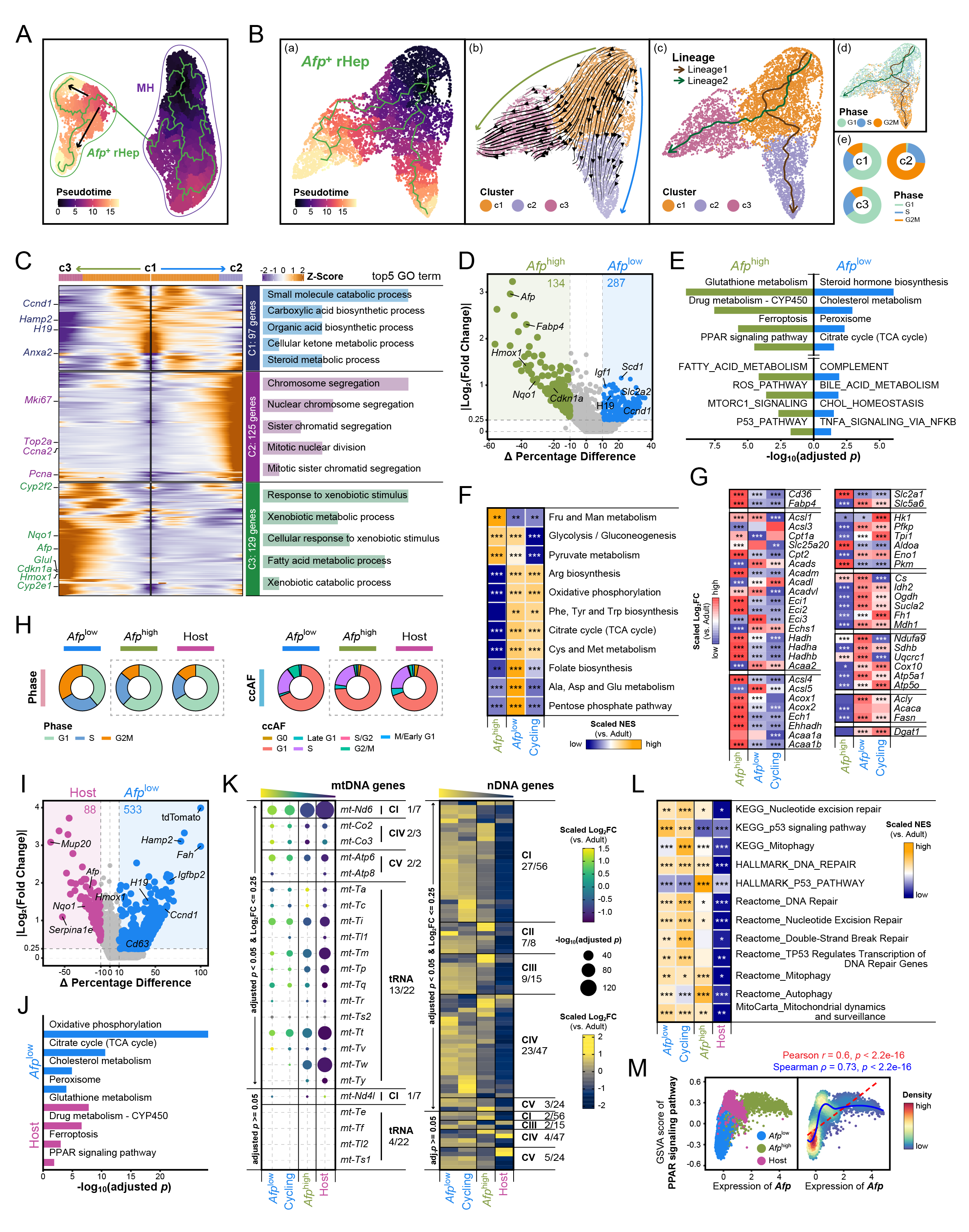


**Figure S5. Transcriptional and metabolic signatures of *Afp*-associated hepatocyte adaptation. Related to Figure 3.** **Additional details are provided in Table S3.**

1. UMAP visualization from **Figure 1B**, colored by pseudotime predicted using Monocle3^[^[^5^](#_ENREF_5)^]^, displaying differentiation trajectories as green lines with black arrows indicating two branches identified in *Afp*^+^ rHeps.
2. UMAP from **Figure 3A**, showcasing pseudotime and RNA velocity analyses. (**a**) Pseudotime (Monocle3) is overlaid on the UMAP (color gradient), with inferred differentiation trajectories shown as green lines. (**b**) RNA velocity streamlines (computed by scVelo^[^[^6^](#_ENREF_6)^]^) are depicted as black curves over a UMAP colored by cell cluster identity. (**c**) UMAP colored by cell cluster identity. (**d**) UMAP colored by cell cycle phase (predicted by Seurat^[^[^7^](#_ENREF_7)^]^). In (**c**) and (**d**), differentiation trajectories inferred by Slingshot^[^[^8^](#_ENREF_8)^]^ are overlaid as two distinct curves. (**e**) Donut charts quantifying the distribution of cell cycle phases within each of the three major clusters.
3. Heatmap (**left**) depicts genes differentially expressed along pseudotime (Monocle2^[^[^9^](#_ENREF_9)^]^, from **Figure 3B**) across both branches. Genes are grouped into modules based on their temporal expression patterns. The bar plot (**right**) shows the top 5 Gene Ontology (GO) terms significantly enriched for each module.
4. Volcano plot based on the percentage difference (x-axis) and the |log2FC| (y-axis) shows the DEGs in *Afp*^high^ vs. *Afp*^low^. Annotation highlights marker genes for each cell type. The counts of DEGs are annotated on the plot.
5. Bar plot displaying the selected KEGG and HALLMARK pathways that are significantly enriched for DEGs in *Afp*^high^ vs. *Afp*^low^ (adjusted *p* < 0.05). The x-axis indicates the -log_10_(adjusted *p*) of the enriched pathways.
6. Heatmap showing the average NES of metabolic pathways for the *Afp*⁺ rHep subpopulations relative to Adult hepatocytes. Asterisks denote significant enrichment (*adjusted *p* < 0.05, **adjusted *p* < 0.01, ***adjusted *p* < 0.001).
7. Heatmap of metabolism-associated genes from **Figure 2M** and **Figure S4J** across *Afp*^+^ rHep subpopulations. Asterisks mark significantly differentially expressed genes relative to Adult hepatocytes (two-sided Wilcoxon rank sum test, *adjusted *p* < 0.05, **adjusted *p* < 0.01, ***adjusted *p* < 0.001).
8. Donut charts display the cell cycle phase distributions predicted by Seurat (**left**) and ccAF^[^[^10^](#_ENREF_10)^]^ (**right**) across three *Afp*^+^ rHep subpopulations.
9. Volcano plot based on the percentage difference (x-axis) and the |log2FC| (y-axis) shows the DEGs in Host vs. *Afp*^low^. Annotation highlights marker genes for each cell type. The counts of DEGs are annotated on the plot.
10. Bar plot displaying the selected KEGG pathways that are significantly enriched for DEGs in Host vs. *Afp*^low^ (adjusted *p*< 0.05). The x-axis indicates the -log_10_(adjusted *p*) of the enriched pathways.
11. Differential expression of electron transport chain (ETC) genes across *Afp*⁺ hepatocyte populations relative to Adult hepatocytes. The dot plot (**left**) displays mitochondrial DNA (mtDNA)-encoded ETC genes, and the heatmap (**right**) depicts nuclear DNA (nDNA)-encoded ETC genes. Genes shown are the complement to those in **Figure 3L**. Color intensity represents the scaled log_2_FC, while dot size corresponds to statistical significance (-log_10_(adjusted *p*)).
12. Heatmap showing the average NES of cellular repair pathways for the *Afp*⁺ hepatocyte population relative to Adult hepatocytes. Asterisks denote significant enrichment (*adjusted *p* < 0.05, **adjusted *p* < 0.01, ***adjusted *p* < 0.001).
13. Scatter plots demonstrate a significant positive correlation between *Afp* expression levels and PPAR signaling pathway activity (gene set variation enrichment analysis, GSVA scores) in *Afp*⁺ hepatocytes (Spearman's *ρ* = 0.73, *p* < 2.2e-16). Cells are colored by populations (**left**) or shaded by density (**right**).


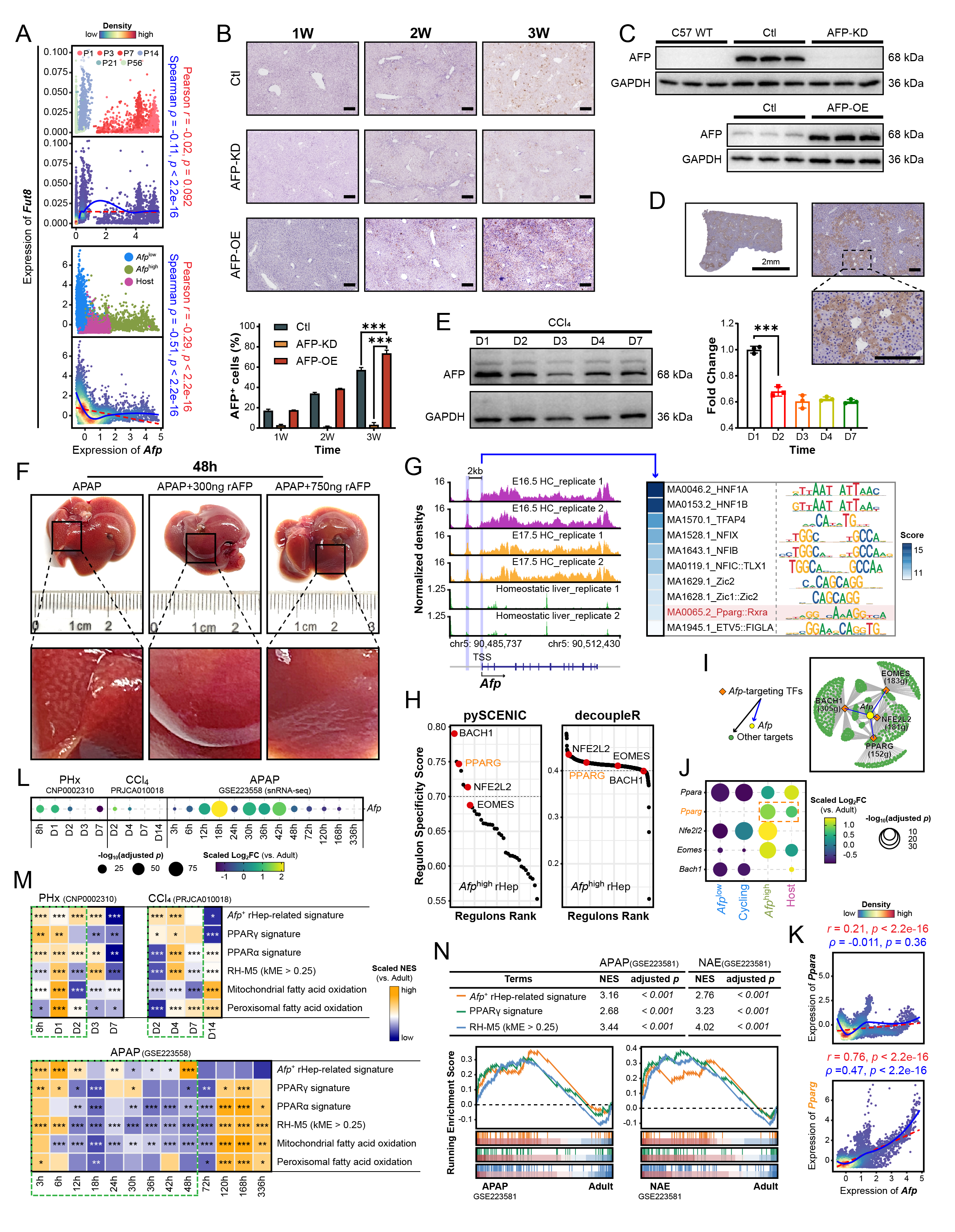


**Figure S6. Expression, regulation, and hepatoprotective potential of AFP in models of liver injury. Related to Figure 4. Additional details are provided in Table S4.**

1. Scatter plots assessing the correlation between *Afp* and *Fut8* expression in postnatal hepatocytes (**left**) and *Afp*⁺ hepatocytes (**right**). Cells are colored by sample groups (**top**) or shaded by density (**bottom**).
2. (Top) Representative AFP IHC staining in liver sections of *Fah*^-/-^ host mice with varying AFP backgrounds (Ctl, AFP-OE, AFP-KD) at 1, 2 and 3 weeks post transplantation with normal adult tdTomato+ hepatocytes. Scale bars: 100 μm. (Bottom) Quantification of AFP⁺ cells, presented as the percentage of AFP⁺ cells per field, in the three recipient groups over time. ****P* < 0.001.
3. Western blot (WB) analysis of AFP protein levels in liver tissue from "C57 WT", "Ctl", "AFP-KD", and "AFP-OE" groups. GAPDH was used as a loading control.
4. (Left) IHC staining of AFP in a CCl_4_-treated liver tissue. Scale bars: 2mm. (Right) Zoom in on the selected field of view. Scale bars: 100 μm.
5. WB analysis of AFP in liver tissues at 1, 2, 3, 4, and 7 days after CCl_4_ administration. GAPDH was used as control.
6. Macroscopic appearance of livers 48 hours after APAP-induced injury.​ Livers from mice treated with APAP (300 mg/kg) alone (Control) show extensive granular necrosis. Co-treatment with low-dose or high-dose recombinant AFP (rAFP) resulted in visibly smoother liver surfaces.
7. (Left) Combined normalized ATAC-seq insertions around the *Afp* locus in hepatocytes from mouse embryonic days E16.5-E17.5^[^[^11^](#_ENREF_11)^]^ and homeostatic liver^[^[^12^](#_ENREF_12)^]^. (Right) Heatmap displaying the enrichment scores for transcription factor (TF) binding motifs identified via motifmatchr within the 400-bp core promoter region (transcription start site, TSS±200 bp) of the *Afp* gene.
8. Rank-ordered plots of regulon specificity (Relative Specificity Score, RSS) for *Afp*^high^ rHeps. Regulons, inferred using pySCENIC^[^[^13^](#_ENREF_13)^]^ (**left**) and decoupleR^[^[^14^](#_ENREF_14)^]^ (**right**), are ranked by their RSS value. Four *Afp*-modulating regulators are highlighted in red.
9. Regulatory network depicting interactions between four *Afp*-modulating transcription factors (TFs; PPARG, NFE2L2, EOMES, and BACH1) and their predicted target genes, inferred from pySCENIC.
10. Dot plot showing the differential expression of *Ppara* and four *Afp*-modulating TF genes (*Pparg*, *Nfe2l2*, *Eomes*, *Bach1*). Each population from **Figure 3G** is individually compared to Adult hepatocytes. Color intensity represents the scaled log_2_FC, while dot size corresponds to statistical significance (-log_10_(adjusted *p*)).
11. Scatter plots show the relationship between *Afp* and *Ppara* expression (**top**, Pearson’s *r* = 0.21, *p* < 2.2e-16) and between *Afp* and *Pparg* expression (**bottom**, Pearson’s *r* = 0.76, *p* < 2.2e-16) in *Afp*⁺ hepatocytes. Cells are shaded by density.
12. Dot plot showing the differential expression of *Afp* in hepatocytes from PHx-, CCl_4_-, and APAP-injured livers during regeneration^[^[^15-17^](#_ENREF_15)^]^, relative to Adult hepatocytes. Color intensity represents the scaled log_2_FC, while dot size corresponds to statistical significance (-log_10_(adjusted *p*)).
13. Heatmap showing the average NES of six gene signatures for hepatocytes from PHx-, CCl4-, and APAP-injured regenerating livers, relative to Adult hepatocytes. Asterisks denote significant enrichment (*adjusted *p* < 0.05, **adjusted *p* < 0.01, ***adjusted *p* < 0.001).
14. GSEA of the ARS, PPARγ, and RH-M5 gene sets, each coded by a distinct color, in APAP vs. Adult hepatocytes (**left**) and NAE vs. Adult hepatocytes (**right**). NES and significance values are tabulated above the plots.


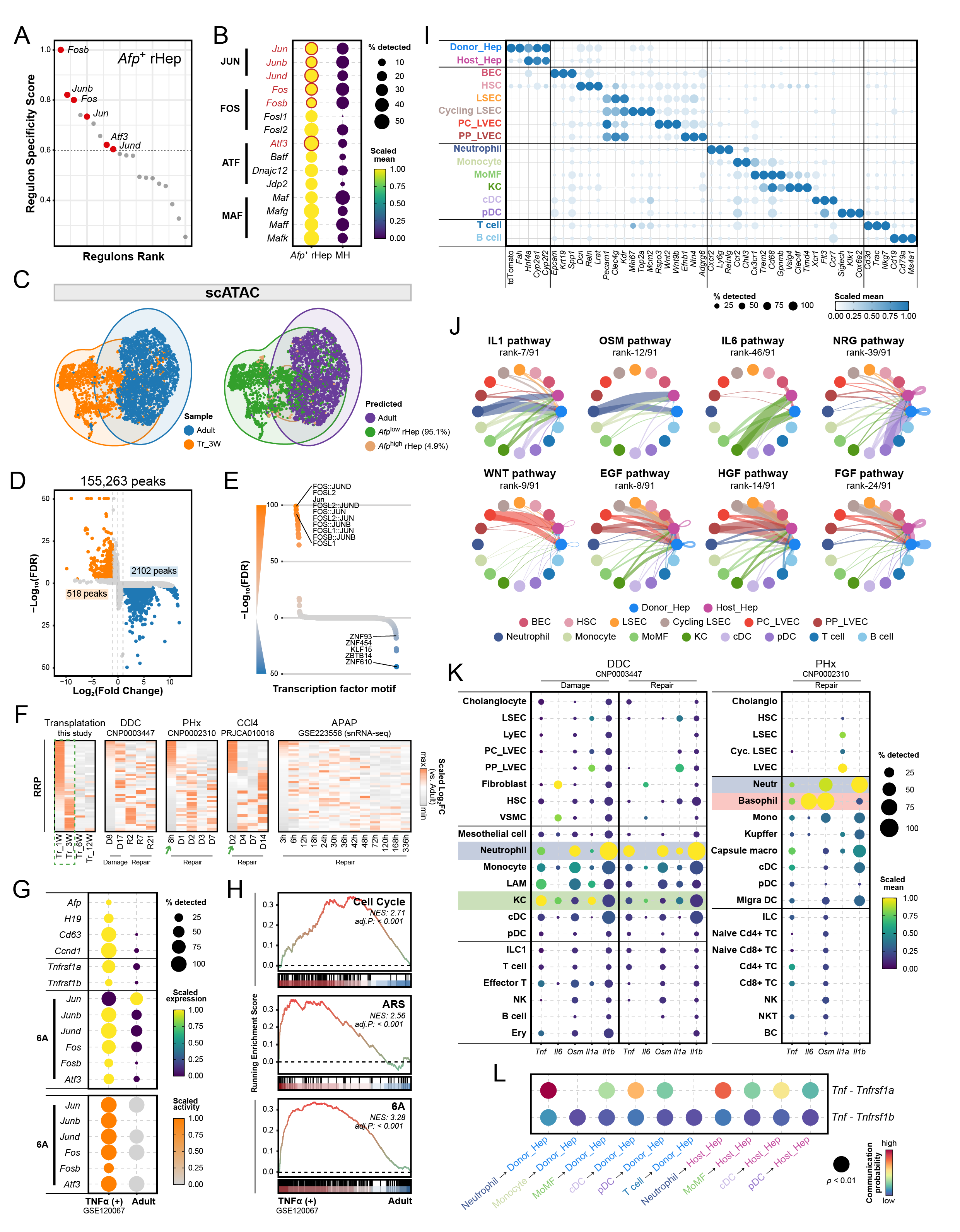


**Figure S7. TNFα-AP-1 axis drives the proliferative-reprogramming of hepatocytes. Related to Figure 5.** **Additional details are provided in Table S5.**

1. Rank-ordered plots of regulon specificity (RSS) for *Afp*^+^ rHeps. pySCENIC-inferred regulons are ranked by their RSS value, with six AP-1 transcription factors highlighted in red.
2. Dot plot showing the scaled expression (color intensity) and cellular percentage (dot size) of AP-1 genes in *Afp*^+^ rHep and MH.
3. UMAP visualization of integrated scATAC-seq data from Tr_3W and Adult hepatocytes. Cells are colored by sample origin (**left**) and inferred cell type based on label transfer from matched scRNA-seq data (**right**). Cells are encircled to highlight group identity and distribution.
4. Volcano plot based on the log_2_FC (x-axis) and the -log_10_(false discovery rate, FDR) (y-axis) shows the differentially accessible peaks (DAPs) in Tr_3W vs. Adult. The counts of DAPs are annotated on the plot.
5. Rank-ordered plot of TF motif enrichment for peaks specific to Tr_3W vs. Adult hepatocytes. Motifs are ranked and colored by the -log_10_(FDR) of their enrichment.
6. Heatmap showing expression of regeneration response program (RRP) genes^[^[^18^](#_ENREF_18)^]^ in transplanted hepatocytes (this study) and hepatocytes from regenerating livers following DDC, PHx, CCl4, and APAP injury (public data)^[^[^15-17^](#_ENREF_15)^,^ [^19^](#_ENREF_19)^]^. Color intensity represents the scaled log_2_FC relative to Adult hepatocytes.
7. Dot plot showing the scaled expression (color intensity) and cellular percentage (dot size) of 6A genes in TNFα-treated (TNFα+)^[^[^20^](#_ENREF_20)^]^ and Adult hepatocytes (**top**). The corresponding decoupleR-inferred activities of 6A TF regulons are shown (**bottom**).
8. GSEA of Cell cycle (**top**), ARS (**middle**), and 6A (**bottom**) gene sets in TNFα+ vs. Adult hepatocytes.
9. Dot plot showing the scaled expression (color intensity) and cellular percentage (dot size) of marker genes for each annotated cell type (as in **Figure 5H**) in scRNA-seq data.
10. Circle plots depict the inferred interaction strength of eight signaling pathways from non-hepatocyte sender cells to hepatocyte receivers (Donor_Hep and Host_Hep).
11. Dot plots show the scaled expression (color intensity) and cellular percentage (dot size) of five cytokine genes across cell types during DDC-induced damage/repair^[^[^19^](#_ENREF_19)^]^ (**left**) and PHx-mediated regeneration^[^[^15^](#_ENREF_15)^]^ (**right**).
12. Bubble plot showing the communication probability of TNF ligand-receptor pairs mediating incoming signals to Donor_Hep and Host_Hep. Statistical significance (*p* < 0.01) for each pair calculated by CellChat^[^[^21^](#_ENREF_21)^]^.


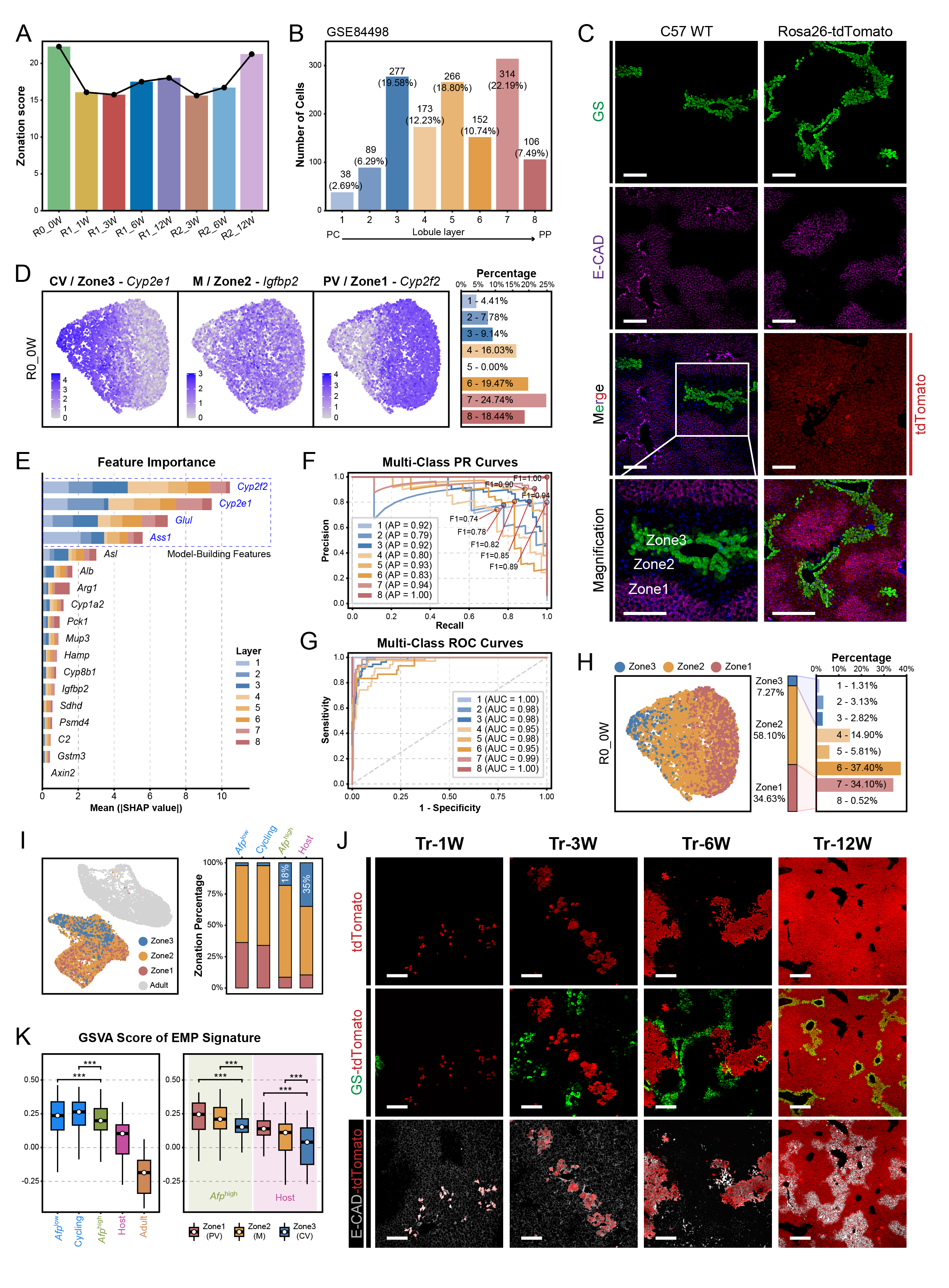


**Figure S8.** **Development of** **a classifier for hepatocyte zonation identification. Related to Figure 6**

1. Bar plot showing the range (max-min) of hepatocyte zonation scores for each of the eight samples.
2. Bar plot showing the number of hepatocytes in each lobular layer (from periportal (PP) to pericentral (PC)) in healthy mouse liver (GSE84498^[^[^22^](#_ENREF_22)^]^).
3. IF staining for GS and E-CAD in livers from WT mice (**left**) and from AAV8-TBG-Cre-injected mice at 3 weeks post-injection (**right**). Staining in the right panel also includes tdTomato. Scale bars: 100 μm.
4. UMAP visualization of zonated gene expression (*Cyp2f2*, *Igfbp2*, *Cyp2e1*) in R0_0W hepatocytes (**left**) and their inferred spatial distribution across lobular layers (1-8) (**right**).
5. SHAP-based feature importance of 18 established zonated genes in XGBoost^[^[^23^](#_ENREF_23)^]^ modeling for lobular layer prediction (GSE84498). Genes selected for final model construction are labeled. Colors denote 8 lobular layers (1-8).

**F-G.** Multi-class prediction performance for lobular layer prediction (GSE84498). Precision-recall (PR) curves (**F**) and receiver operating characteristic (ROC) curves (**G**) are shown, both color-coded by the 8 lobular layers (1-8). Per-class metrics (AP, F1, AUC) are labeled.

1. Analysis of model-predicted zonation in R0_0W hepatocytes: UMAP visualization colored by predicted zone (**left**), composition of zonation proportions (**middle**), and inferred spatial distribution across lobular layers (**right**).
2. (**Left**) The same UMAP projection of hepatocytes from **Figure 3F**, recolored by model-predicted lobular zonation (Zone 1-3). (**Right**) Stacked bar plot showing the proportional distribution of zonation labels across the non-adult cell groups defined in **Figure 3F**.
3. IF staining for tdTomato (**top**) and GS (**middle**) or E-CAD (**bottom**) in host livers at 4 timepoints post-transplantation (Tr-1W, 3W, 6W and 12W). **Figure 6C** is merged diagrams with 3 colors of **Figure S8K**. Scale bars:100 μm.
4. (**Left**) Box plots showing the distribution of EMP pathway activity scores (GSVA) across the five hepatocyte groups defined in **Figure 3F**. (**Right**) Box plots displaying the distribution of EMP scores in *Afp*^high^ and Host cells, stratified by model-predicted lobular zonation established in **Figure S8I**. Statistical comparisons (two-sided Wilcoxon rank-sum test) were performed between the indicated groups, ***adjusted *p* < 0.001.

**Supplementary Tables**

**Supplementary Table 1.** Related to Figure 1 and Figure S2.

**Supplementary Table 2.** Related to Figure 2 and Figure S4.

**Supplementary Table 3.** Related to Figure 3 and Figure S5.

**Supplementary Table 4.** Related to Figure 4 and Figure S6.

**Supplementary Table 5.** Related to Figure 5 and Figure S7.

**Supplementary Table 6.** Related to Figure 6.

**Supplementary Table 7.** Gene sets used in this study.

**Supplementary Table 8.** Key resources table.

Supplementary References

[1] L. Machado, P. Geara, J. Camps, M. Dos Santos, F. Teixeira-Clerc, J. Van Herck, H. Varet, R. Legendre, J. M. Pawlotsky, M. Sampaolesi, T. Voet, P. Maire, F. Relaix, P. Mourikis, *Cell Stem Cell* **2021**, *28* (6), 1125, <https://doi.org/10.1016/j.stem.2021.01.017>.

[2] L. Yang, X. Wang, J. X. Zheng, Z. R. Xu, L. C. Li, Y. L. Xiong, B. C. Zhou, J. Gao, C. R. Xu, *Dev Cell* **2023**, *58* (19), 1996, <https://doi.org/10.1016/j.devcel.2023.07.006>.

[3] L. Li, L. Cui, P. Lin, Z. Liu, S. Bao, X. Ma, H. Nan, W. Zhu, J. Cen, Y. Mao, X. Ma, L. Jiang, Y. Nie, F. Ginhoux, Y. Li, H. Li, L. Hui, *Cell Stem Cell* **2023**, *30* (3), 283, <https://doi.org/10.1016/j.stem.2023.01.009>.

[4] N. Alghamdi, W. Chang, P. Dang, X. Lu, C. Wan, S. Gampala, Z. Huang, J. Wang, Q. Ma, Y. Zang, M. Fishel, S. Cao, C. Zhang, *Genome Res* **2021**, *31* (10), 1867, <https://doi.org/10.1101/gr.271205.120>.

[5] J. Cao, M. Spielmann, X. Qiu, X. Huang, D. M. Ibrahim, A. J. Hill, F. Zhang, S. Mundlos, L. Christiansen, F. J. Steemers, C. Trapnell, J. Shendure, *Nature* **2019**, *566* (7745), 496, <https://doi.org/10.1038/s41586-019-0969-x>.

[6] V. Bergen, M. Lange, S. Peidli, F. A. Wolf, F. J. Theis, *Nat Biotechnol* **2020**, *38* (12), 1408, <https://doi.org/10.1038/s41587-020-0591-3>.

[7] Y. Hao, S. Hao, E. Andersen-Nissen, W. M. Mauck, 3rd, S. Zheng, A. Butler, M. J. Lee, A. J. Wilk, C. Darby, M. Zager, P. Hoffman, M. Stoeckius, E. Papalexi, E. P. Mimitou, J. Jain, A. Srivastava, T. Stuart, L. M. Fleming, B. Yeung, A. J. Rogers, J. M. McElrath, C. A. Blish, R. Gottardo, P. Smibert, R. Satija, *Cell* **2021**, *184* (13), 3573, <https://doi.org/10.1016/j.cell.2021.04.048>.

[8] K. Street, D. Risso, R. B. Fletcher, D. Das, J. Ngai, N. Yosef, E. Purdom, S. Dudoit, *BMC Genomics* **2018**, *19* (1), 477, <https://doi.org/10.1186/s12864-018-4772-0>.

[9] X. Qiu, Q. Mao, Y. Tang, L. Wang, R. Chawla, H. A. Pliner, C. Trapnell, *Nat Methods* **2017**, *14* (10), 979, <https://doi.org/10.1038/nmeth.4402>.

[10] S. A. O'Connor, L. Garcia, R. Hoover, A. P. Patel, B. B. Bartelle, J. P. Hugnot, P. J. Paddison, C. L. Plaisier, *bioRxiv* **2025**, <https://doi.org/10.1101/2024.04.16.589816>.

[11] L. Yang, X. Wang, X. X. Yu, L. Yang, B. C. Zhou, J. Yang, C. R. Xu, *Dev Cell* **2023**, *58* (18), 1688, <https://doi.org/10.1016/j.devcel.2023.07.002>.

[12] C. Liu, M. Wang, X. Wei, L. Wu, J. Xu, X. Dai, J. Xia, M. Cheng, Y. Yuan, P. Zhang, J. Li, T. Feng, A. Chen, W. Zhang, F. Chen, Z. Shang, X. Zhang, B. A. Peters, L. Liu, *Sci Data* **2019**, *6* (1), 65, <https://doi.org/10.1038/s41597-019-0071-0>.

[13] B. Van de Sande, C. Flerin, K. Davie, M. De Waegeneer, G. Hulselmans, S. Aibar, R. Seurinck, W. Saelens, R. Cannoodt, Q. Rouchon, T. Verbeiren, D. De Maeyer, J. Reumers, Y. Saeys, S. Aerts, *Nat Protoc* **2020**, *15* (7), 2247, <https://doi.org/10.1038/s41596-020-0336-2>.

[14] I. M. P. Badia, J. Vélez Santiago, J. Braunger, C. Geiss, D. Dimitrov, S. Müller-Dott, P. Taus, A. Dugourd, C. H. Holland, R. O. Ramirez Flores, J. Saez-Rodriguez, *Bioinform Adv* **2022**, *2* (1), vbac016, <https://doi.org/10.1093/bioadv/vbac016>.

[15] J. Xu, P. Guo, S. Hao, S. Shangguan, Q. Shi, G. Volpe, K. Huang, J. Zuo, J. An, Y. Yuan, M. Cheng, Q. Deng, X. Zhang, G. Lai, H. Nan, B. Wu, X. Shentu, L. Wu, X. Wei, Y. Jiang, X. Huang, F. Pan, Y. Song, R. Li, Z. Wang, C. Liu, S. Liu, Y. Li, T. Yang, Z. Xu, W. Du, L. Li, T. Ahmed, K. You, Z. Dai, L. Li, B. Qin, Y. Li, L. Lai, D. Qin, J. Chen, R. Fan, Y. Li, J. Hou, M. Ott, A. D. Sharma, T. Cantz, A. Schambach, K. Kristiansen, A. P. Hutchins, B. Göttgens, P. H. Maxwell, L. Hui, X. Xu, L. Liu, A. Chen, Y. Lai, M. A. Esteban, *Nat Genet* **2024**, *56* (5), 953, <https://doi.org/10.1038/s41588-024-01709-7>.

[16] S. Wang, X. Wang, Y. Shan, Z. Tan, Y. Su, Y. Cao, S. Wang, J. Dong, J. Gu, Y. Wang, *Cell Stem Cell* **2024**, *31* (3), 341, <https://doi.org/10.1016/j.stem.2024.01.013>.

[17] K. P. Matchett, J. R. Wilson-Kanamori, J. R. Portman, C. A. Kapourani, F. Fercoq, S. May, E. Zajdel, M. Beltran, E. F. Sutherland, J. B. G. Mackey, M. Brice, G. C. Wilson, S. J. Wallace, L. Kitto, N. T. Younger, R. Dobie, D. J. Mole, G. C. Oniscu, S. J. Wigmore, P. Ramachandran, C. A. Vallejos, N. O. Carragher, M. M. Saeidinejad, A. Quaglia, R. Jalan, K. J. Simpson, T. J. Kendall, J. A. Rule, W. M. Lee, M. Hoare, C. J. Weston, J. C. Marioni, S. A. Teichmann, T. G. Bird, L. M. Carlin, N. C. Henderson, *Nature* **2024**, *630* (8015), 158, <https://doi.org/10.1038/s41586-024-07376-2>.

[18] W. Wang, C. K. Hu, A. Zeng, D. Alegre, D. Hu, K. Gotting, A. Ortega Granillo, Y. Wang, S. Robb, R. Schnittker, S. Zhang, D. Alegre, H. Li, E. Ross, N. Zhang, A. Brunet, A. Sánchez Alvarado, *Science* **2020**, *369* (6508), <https://doi.org/10.1126/science.aaz3090>.

[19] B. Wu, X. Shentu, H. Nan, P. Guo, S. Hao, J. Xu, S. Shangguan, L. Cui, J. Cen, Q. Deng, Y. Wu, C. Liu, Y. Song, X. Lin, Z. Wang, Y. Yuan, W. Ma, R. Li, Y. Li, Q. Qian, W. Du, T. Lai, T. Yang, C. Liu, X. Ma, A. Chen, X. Xu, Y. Lai, L. Liu, M. A. Esteban, L. Hui, *Nat Genet* **2024**, *56* (5), 938, <https://doi.org/10.1038/s41588-024-01687-w>.

[20] W. C. Peng, C. Y. Logan, M. Fish, T. Anbarchian, F. Aguisanda, A. Álvarez-Varela, P. Wu, Y. Jin, J. Zhu, B. Li, M. Grompe, B. Wang, R. Nusse, *Cell* **2018**, *175* (6), 1607, <https://doi.org/10.1016/j.cell.2018.11.012>.

[21] S. Jin, M. V. Plikus, Q. Nie, *Nat Protoc* **2025**, *20* (1), 180, <https://doi.org/10.1038/s41596-024-01045-4>.

[22] K. B. Halpern, R. Shenhav, O. Matcovitch-Natan, B. Toth, D. Lemze, M. Golan, E. E. Massasa, S. Baydatch, S. Landen, A. E. Moor, A. Brandis, A. Giladi, A. S. Avihail, E. David, I. Amit, S. Itzkovitz, *Nature* **2017**, *542* (7641), 352, <https://doi.org/10.1038/nature21065>.

[23] T. Chen, C. Guestrin, presented at *Proceedings of the 22nd ACM SIGKDD International Conference on Knowledge Discovery and Data Mining*, San Francisco, California, USA, **2016**.
